# Autophagy in plants

**DOI:** 10.1080/27694127.2024.2395731

**Published:** 2024-10-15

**Authors:** Morten Petersen, Tamar Avin-Wittenberg, Diane C. Bassham, Yasin Dagdas, Chudi Fan, Alisdair R. Fernie, Liwen Jiang, Divya Mishra, Marisa S. Otegui, Eleazar Rodriguez, Daniel Hofius

**Affiliations:** a Department of Biology, Faculty of Science, Copenhagen University, Copenhagen, Denmark; b Department of Plant and Environmental Sciences, The Alexander Silberman Institute of Life Sciences, The Hebrew University of Jerusalem, Givat Ram, Jerusalem, Israel; c Department of Genetics, Development and Cell Biology, Iowa State University, Ames, IA, USA; d Gregor Mendel Institute, Austrian Academy of Sciences, Vienna BioCenter, Vienna, Austria; e School of Life Sciences, Centre for Cell & Developmental Biology and State Key Laboratory of Agrobiotechnology, The Chinese University of Hong Kong, Shatin, New Territories, Hong Kong, China; f Max Planck Institute of Molecular Plant Physiology, Potsdam-Golm, Germany; g Department of Botany, University of Wisconsin, Madison, WI, USA; h Center for Quantitative Cell Imaging, University of Wisconsin, Madison, WI, USA; i Department of Plant Biology, Uppsala BioCenter, Swedish University of Agricultural Sciences and Linnean Center for Plant Biology, Uppsala, Sweden

**Keywords:** Plant autophagy, regulation and signalling, endomembrane trafficking, quality control, cargo receptors, development, stress tolerance, immunity, metabolism, crop improvement

## Abstract

Autophagy is a process of cellular self-eating, which allows organisms to eliminate and recycle unwanted components and damaged organelles to maintain cellular homeostasis. It is an important process in the development of eukaryotic organisms. Autophagy plays a critical role in many physiological processes in plants such as nutrient remobilization, cell death, immunity, and abiotic stress responses. Autophagy thus represents an obvious target for generating resilient crops. During plant development, autophagy is also implicated in the differentiation and maturation of various cell types and plant organs, including root cap cells, tracheary elements, gametes, fruits and seeds. Here, we review our current understanding and recent advances of plant autophagy including insight into autophagy regulation and signaling as well as autophagosome membrane biogenesis. In addition, we describe how autophagy contributes to development, metabolism, biotic and abiotic stress tolerance and where the autophagic field is heading in terms of applied research for crop improvement.

## Introduction

Autophagy is a highly conserved process in which cells deliver cytoplasmic components to their lysosomes or vacuoles for recycling or storage, particularly under stressful conditions. This process is crucial for maintaining cellular homeostasis and plays a key role in responses to biotic and abiotic stress, nutrient deprivation, and development. In plants, two main autophagy modalities have been identified: macroautophagy and microautophagy [1,2]. Macroautophagy is the best studied of the two pathways and is commonly referred to simply as autophagy. Activation of macroautophagy results in the formation of a cup-shaped double-membrane structure, called the phagophore, that typically develops from the endoplasmic reticulum (ER) in plants. As the phagophore extends and closes, it sequesters cytoplasmic material in an autophagosome. Autophagosomes loaded with cytoplasmic cargo such as organelles, ribosomes, and protein aggregates fuse with the vacuolar membrane, releasing the inner core or macroautophagic body for breakdown by hydrolytic vacuolar enzymes. In microautophagy, the cytoplasmic proteins or organelles closely associate with the vacuolar membrane, which directly engulf the cargo by invagination, forming a microautophagic body that is then degraded or stored in the vacuolar lumen. A third and less known form of autophagy reported in plants is mega-autophagy, in which the vacuole bursts and releases the hydrolytic enzyme to degrade the entire cytoplasm, as the final steps of developmental programmed cell death in cell types such as tracheary elements in the xylem [3].

In recent years, substantial progress has been achievd in the understanding of the mechanisms and functions of autophagy in plants, and it is now evident that autophagy is part of most if not all aspects of the plant’s life cycle. Primary discoveries are therefore of outstanding interest for the development of many agricultural traits (reviewed in [4-6]) and have also strong implications for the wider field of autophagy [7-9]. In this review, we will give insight into the recent advances of autophagy research in plants by focussing on the regulation of the autophagy machinery, autophagosome biogenesis and selective autophagy processes. We will further highlight the emerging roles of autophagy in metabolism, development, immunity, and abiotic stress responses, and outline the growing efforts to dissect and utilize autophagy mechanisms for the improvement of productivity and resilience in crop species.

## Autophagy core machinery – regulation and signaling

**Figure 1.**
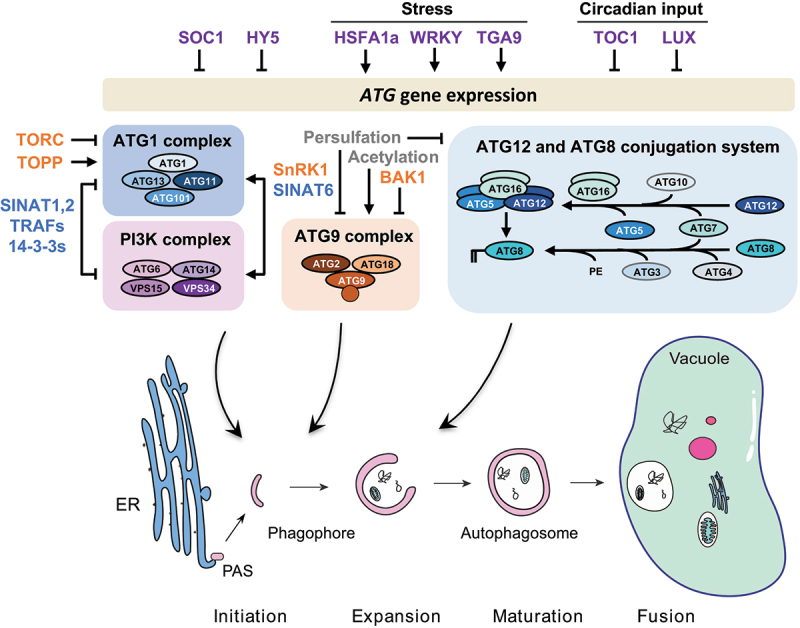
Overview of the autophagy pathway and the major mechanisms regulating the autophagy core machinery in plants. Plant genomes contain more than 40 *ATG* genes that encode the functional units of the autophagy machinery to drive autophagosome biogenesis. Autophagosomes are initiated at ER-localised preautophagosomal structures (phagophore assembly sites, PAS) and mature after recruitment of cellular content to the expanding phagophore. Upon autophagosomal fusion with the vacuole, the sequestered cargo is released for lytic degradation and subsequent recycling. Transcription factors (purple) activate or repress *ATG* genes in response to environmental or endogenous cues. Protein kinases and phosphatases (orange) modify autophagy core components to control their activity. Post-translational modifications (grey) of autophagy proteins by persulfidation inhibit or by acetylation activate autophagy. Stability of the autophagy machinery is modulated by ubiquitination and subsequent degradation (blue). See text for details.

The core genes encoding the autophagy machinery, termed *ATG* (autophagy-related) genes, were initially identified in yeast [10-12] and are highly conserved throughout eukaryotes, including plants [7] (Figure 1). Autophagy is initiated upon activation of the ATG1 kinase complex [13], consisting of the ATG1 catalytic subunit together with additional ATG13, ATG11 and ATG101 subunits, which phosphorylates and activates downstream autophagy components. A phosphatidylinositol 3-kinase complex (PI3K) produces phosphatidylinositol 3-phosphate (PI3P) at the phagophore [14], recruiting PI3P binding proteins including ATG18 [15]. Expansion and maturation of the phagophore requires delivery of lipids by the lipid scramblase ATG9 [16] and lipid transfer protein ATG2 [17], and two ubiquitin-like conjugation cascades culminating in the conjugation of the ubiquitin-related protein ATG8 to phosphatidylethanolamine (PE) on the autophagosome membrane [18]. The critical role of the autophagy pathway in controlling development, stress responses and cell death necessitates tight control of autophagy activity in response to environmental and hormonal cues. This regulation is most prominently by post-translational modification of the core autophagy machinery, although transcriptional regulation of *ATG* genes is also important under a number of conditions (Figure 1).

### Transcriptional regulation of autophagy genes

Some knowledge is now being accumulated on transcriptional regulation of *ATG* genes, although it is certain that much more is yet to be discovered. Earlier work in tomato showed that heat and drought stress induce autophagy, and transcription factors that upregulate *ATG* genes are key components of the stress response, allowing tolerance of these conditions. These include HSFA1a (heat shock factor A1a), a transcription factor that activates genes encoding proteins of the autophagy core machinery during drought [19], including *ATG10* and *ATG18*, and WRKY-family transcription factors, which upregulate several *ATG* genes during heat stress [20].

More recently, several transcription factors that regulate *ATG* gene expression, and therefore autophagy, have been identified in Arabidopsis. For example, the MADS box-family transcription factor SOC1 (suppressor of overexpression of constans 1) has been shown to repress the expression of several *ATG* genes. A *soc1* mutant has increased autophagy and tolerance of fixed-carbon starvation, and SOC1 may repress autophagy to prevent its inappropriate activation [21]. Similarly, HY5 (elongated hypocotyl 5) represses the expression of a subset of *ATG* genes, and a *hy5* mutant has increased autophagy. HY5 acts by recruiting HDA9 (histone deacetylase 9) to *ATG* genes to suppress their expression, revealing that autophagy can also be regulated epigenetically [22]. HY5 is degraded by the proteasome in the dark or upon nitrogen deficiency, leading to release of its repressive activity and activation of autophagy in response to these stresses [22].

In plants, ATG8 is encoded by a multiple gene family, with the ATG8 isoforms having distinct spatial and temporal expression patterns. Numerous transcription factors that bind to *ATG8* promoters were identified by yeast 1-hybrid screening, and the bZIP family factor TGA9 (TGACG motif-binding protein 9) was confirmed as a positive regulator of autophagy via the upregulation of a range of *ATG* genes [23].

Autophagy has recently been shown to be controlled by the circadian clock, and two circadian-related transcription factors have been identified as negative regulators of *ATG* gene expression. TOC1 (timing of cab expression 1) is a core oscillator component, and directly binds to the promoters of several autophagy genes to repress their expression [24]. In addition, LUX (lux arrhythmo) represses the expression of a different set of *ATG* genes and prevents rhythmic autophagy activity [25].

Each of these transcription factors appears to regulate a distinct subset of *ATG* genes, and the rationale behind this complex regulation is unclear. Whether each *ATG* promoter binds a different set of transcription factors to provide gene-specific regulation of its transcript levels, or whether this reflects a limitation of the experimental approaches used, is not yet known.

### Post-translational regulation of core autophagy components

#### ATG1 complex

Autophagosome formation is initiated by the ATG1 protein kinase complex. In Arabidopsis, ATG1, in complex with ATG13, activates autophagy, and in turn these proteins are packaged into autophagosomes and degraded by autophagy during starvation [26]. This feedback presumably controls the extent of autophagy activation and prevents excessive autophagic degradation of cell contents. Activity of the ATG1 complex is under the negative control of the protein kinase TORC (target of rapamycin complex) [13], which regulates growth and metabolism in response to nutrient conditions [27,28]. A phosphoproteomic analysis of Arabidopsis cell culture in the presence or absence of a TORC inhibitor identified putative TORC phosphorylation targets [29]. ATG13 and ATG1 were found as likely major TOR substrates, and their sites of phosphorylation identified; phosphorylation at these sites may regulate autophagy activation. A second nutrient and energy sensing kinase, SnRK1 (snf1-related kinase 1), also phosphorylates ATG1 [30] . SnRK1 is a positive regulator of autophagy [31], and therefore phosphorylation by TORC and SnRK1 may act antagonistically to determine the extent of autophagy activation. Dephosphorylation of ATG13 by TOPP (type one protein phosphatase) is also required for autophagy induction, probably by counteracting phosphorylation by the negative regulator TORC. TOPP is encoded by a large gene family, and mutation of multiple members of this family leads to decreased autophagy. TOPP interacts with and can dephosphorylate ATG13, in turn leading to increased ATG1 complex formation and activation of autophagy [32].

While ATG1 and ATG13 can be degraded by autophagy upon induction [26], they are also degraded by the 26S proteasome during prolonged starvation and recovery [33]. This degradation is mediated by the SINAT (seven in absentia of *Arabidopsis thaliana*) 1 and 2 E3 RING-type ubiquitin ligases, together with the TRAF (tumor necrosis factor receptor-associated factor) adaptors, which ubiquitinate ATG13 and regulate ATG1 complex activity. The authors propose that appropriate autophagy levels are maintained upon prolonged starvation by degradation of ATG13 via SINAT1 and 2. In contrast, SINAT6, which has a truncated RING domain and is catalytically inactive, interacts with ATG13 and suppresses its ubiquitination and degradation, thus promoting autophagy [33]. Autophagy therefore is controlled by competition between SINAT1 and 2 as negative regulators and SINAT6 as a positive regulator. Further complexity is added by a recent report of a role for 14-3-3 proteins in ATG13 stability [34]. 14-3-3 proteins bind both to SINAT1 and ATG13, and are required for the degradation of ATG13 upon ubiquitination by SINAT1. 14-3-3 mutants have increased ATG1 and ATG13 levels and increased starvation tolerance, placing them as negative regulators of autophagy. Consistent with the general function of 14-3-3 proteins in binding to phosphorylated peptide sequences [35], phosphorylation of ATG13 is required for its interaction with 14-3-3, and for ATG13 ubiquitination and ATG1 complex dissociation.

#### PI3K complex

The PI3K complex produces PI3-phosphate (PI3P) at expanding autophagosomes, which enables recruitment of other autophagy components to the membrane. The stability and phosphorylation state of the non-catalytic subunit ATG6 regulates the complex. SINAT1 and 2, along with TRAF adaptors, control ATG6 stability similarly to that of ATG13 described above. SINAT1 and 2 ubiquitinate and degrade ATG6 upon starvation, in a reaction that also requires TRAFs, whereas SINAT6 has the opposite effect [36]. The SINATs therefore coordinately regulate the activities of the ATG1 and PI3K complexes to maintain the required autophagy levels. ATG6 is phosphorylated by SnRK1 during prolonged fixed-carbon starvation [37,38], and this appears to directly activate autophagy, with no requirement for ATG1 complex activation under these conditions [38]. Multiple initiation pathways for autophagy may therefore exist, dependent on the precise environmental conditions to which the plants are exposed.

PI3P at the nascent phagophore recruits the PI3P-binding protein ATG18, which functions in membrane elongation [39]. Arabidopsis ATG18a can be phosphorylated by the BAK1 (BRI1 [brassinosteroid insensitive 1]-associated kinase 1), suppressing autophagosome formation and decreasing resistance to necrotrophic pathogens [40,41], although whether it acts under other conditions is not yet known. ATG18a is also modified by persulfidation, again suppressing autophagy. Persulfidation increases binding of ATG18a to PI3P, leading to production of larger but fewer autophagosomes upon ER stress [42]. By contrast, acetylation of ATG18a by the acetyltransferase HOOKLESS1 increases autophagy, and blocking this acetylation inhibits binding to PI3P [43]. Post-translational modification of ATG18a therefore may control the release of ATG18a from the membrane, determining the size versus number of autophagosomes produced and thus, regulating the extent of autophagic degradation.

#### ATG8-PE conjugation

A substantial fraction of the core conserved ATG factors function in ubiquitin-like conjugation reactions that ultimately result in conjugation of ATG8 to PE in the phagophore membrane, which is required for autophagosome expansion and maturation [7]. The protease ATG4 is involved in two steps of this pathway, maturation of ATG8 by removing its C-terminus after synthesis and removal of ATG8 from the outer membrane of the autophagosome prior to fusing with the vacuole [44]. ATG4 was isolated in a screen for proteins persulfidated in response to abscicic acid (ABA), with persulfidation occurring at the proteolytic active site Cys. This modification reversibly inactivated the protease, thereby inhibiting autophagy [45]. The authors hypothesized that ATG4 is persulfidated in normal growth conditions, keeping autophagy activity low. Under stress conditions, a decrease in ATG4 persulfidation increases its activity, allowing autophagy activation via lipidation of ATG8. The redox-sensitive active site of ATG4 also makes it a candidate for redox regulation. While the role of redox regulation in vivo in response to stress in plants is not yet clear, ATG4 protease activity has been shown to be reversibly inhibited by H_2_O_2_ [46], and in the green alga *Chlamydomonas reinhardtii*, ATG4 is activated by disulfide bond reduction, as disulfide bond formation inhibits its activity [47].

Other mechanisms of regulation of the ubiquitin-like conjugation pathways include interactions with other proteins, regulation of protein stability, and regulation of ATG8 lipidation. For example, CML24 (calmodulin-like 24), a calmodulin-related protein, interacts with ATG4b and controls its activity [48]; GAPC (cytosolic glyceraldehyde-3-phosphate dehydrogenase) interacts with ATG3 and regulates autophagy activity [49,50], in a phosphatidic acid-dependent manner [51]; phospholipase Dɛ hydrolyzes ATG8-PE, although paradoxically this appears to activate autophagy [52]; ACBP3 (acyl-Co-A binding protein 3) may compete with ATG8 for binding to phosphatidylethanolamine (PE) [53]; COST1 (constitutively stressed 1) interacts with ATG8 and regulates its stability [54]. The coordination between these modes of regulation is unclear, and much remains to be determined regarding how they interact to control the overall activity of the autophagy pathway.

In summary, autophagy in plants is tightly regulated in response to environmental and developmental cues, and this regulation often involves the post-translational modification of core components of the autophagy machinery to control their activity. Transcriptional regulation and degradation of autophagy proteins are also critical in activation of autophagy and in modulating its activity upon prolonged stress. How these regulatory mechanisms work together to enable growth, development and stress tolerance remains an exciting area of research.

## Autophagosome biogenesis and endomembrane trafficking

**Figure 2.**
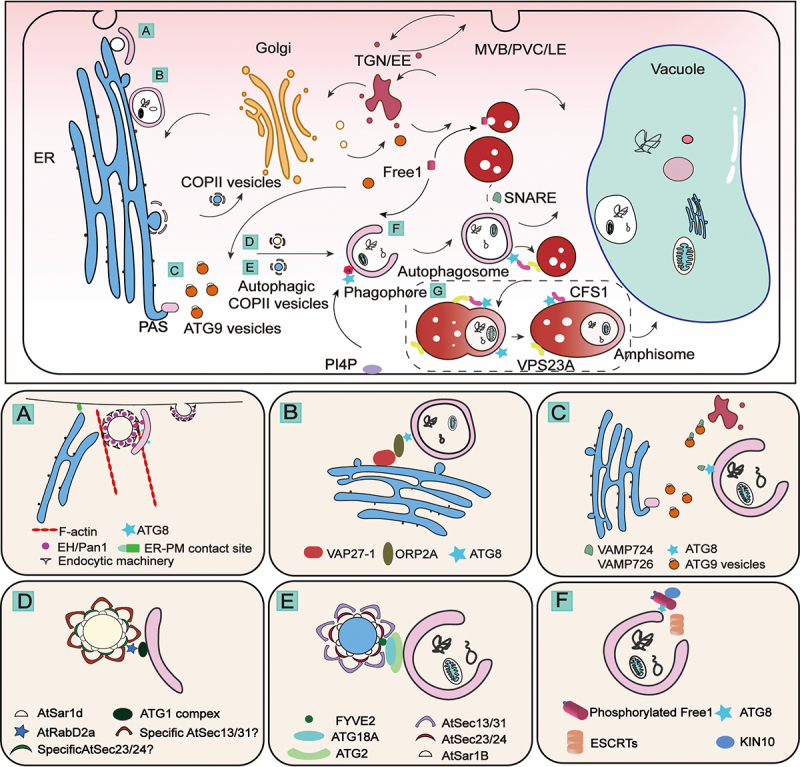
Updated interactions between the endomembrane system and autophagosome biogenesis in plants. The plant endomembrane system contains multiple membrane-bound organelles including the endoplasmic reticulum (ER), the Golgi apparatus (GA), trans-Golgi network/early endosome (TGN/EE), multivesicular body/prevacuolar compartment/late endosome (MVB/PVC/LE), and vacuole. The endomembrane system contributes to autophagosome biogenesis via multiple new mechanisms or pathways (A-F) for subsequent autophagosome-vacuole fusion and degradation in plants, with mechanistic details highlighted in the corresponding enlarged boxes shown below. (A) Autophagosome biogenesis is regulated by AtEH/Pan1, F-actin and endocytic machinery at the ER-PM contact site (EPCS) for the degradation of endocytic components in Arabidopsis. (B) The Arabidopsis ORP2A coordinates with both the ER residential VAP27-1 and ATG8 on the autophagosome to mediate ER-autophagosomal MCS for autophagosome biogenesis. (C) The Arabidopsis SNARE family proteins AtVAMP724/AtVAMP726 regulate the trafficking of ATG9 vesicles. (D) Distinct AtSAR1d-positive COPII population is formed to regulate autophagic flux via the AtSAR1D-AtRABD2a nexus. FYVE2 interacts with AtSar1b and ATG18-ATG2 complex to regulate autophagosome biogenesis. (F) Phosphorylated FREE1 coordinates with both the ESCRT and ATG machinery to mediate autophagosome closure in Arabidopsis. (G) CFS1 interacts with ATG8 and VPS23A in mediating autophagosome-MVB fusion to facilitate the formation of amphisome.

During autophagosome biogenesis, internal and external signals such as nutrient deprivation, stresses, abnormal protein aggregates or damaged organelles trigger the initiation of autophagosomes by hierarchical ATG complex recruitment [55] (Figure 1 and 2). An “autophagosome precursor” (preautophagosomal structure/phagophore assembly site [PAS]), which later transforms into the phagophore after membrane nucleation, is generated upon autophagy induction from the ER with the recruitment of the ATG1/ULK1 complex [56], ATG9 vesicles [57], and COPII (coat protein complex II) vesicles [58]. Subsequently, the phagophore matures into the autophagosome after PI3K-complex-mediated membrane expansion [59] and later membrane closure depending on ATG8 in yeast or LC3 (light chain 3)/GABARAP (GABA type A receptor-associated protein) in mammals (herafter referred to as ATG8 proteins) [60]. The last step of autophagic flux is the autophagosome-lysosome/vacuole fusion mediated by specific soluble SNARE (N-ethylmaleimide-sensitive factor attachment protein receptor) proteins leading to degradation and recycling of cargos [61].

In recent years, plant-unique components and mechanisms involved in autophagosome biogenesis have also been revealed, including membrane contact sites (MCSs) and functionally diverse COPII components participating in autophagosome biogenesis. Crosstalk between the endocytic pathway and autophagosome closure linked by the plant-unique ESCRT (endosomal sorting complex required for transport) component FREE1 (FYVE domain protein required for endosomal sorting 1), and autophagy-related plant SNARE proteins as well as their interactions with ATG9 in autophagosome maturation has also been established.

### Membrane contact sites in plant autophagosome biogenesis

MCSs are formed between heterologous or homologous membranes of two closely apposed organelles (10nm-30nm) [62]. ER forms MCSs with other organelles to mediate dynamic lipid or membrane exchange and trafficking activities [62,63]. In mammalian cells, ER-organelle contacts are crucial for autophagosome biogenesis. For example, ER-mitochondria contact sites participate in autophagosome formation [64]. The ER-localized protein STX17 (syntaxin 17) recruits VPS34 (vacuolar protein sorting 34) PI3K complex to ER-mitochondria MCS, where the PI3P is generated for autophagosome formation [64]. In contrast, the ER-plasma membrane (PM) contact sites (EPCSs) are involved in autophagosome assembly, where extended synaptotagmins (E-syts) form complexes with VPS34 and VMP1 (vacuolar membrane protein 1) for autophagosome formation [65].

The Arabidopsis EH proteins (EH1/Pan1) are components of the TPLATE complex (TPC) that regulate endocytosis and actin-mediated autophagy (Figure 2A). EH1/Pan1 localizes on both PM and autophagosomes, interacting with the ER-resident protein VAP27-1 (VAMP [vesicle-associated membrane protein]-associated protein 27-1] as a complex to bridge the EPCSs and regulate autophagosome biogenesis. Upon nutrient starvation conditions, F-actin and the endocytic machinery, including the TPC, AP-2 (adaptor protein-2) protein and clathrin, are recruited to autophagosomes by EH1/Pan1 at the EPCSs, and contribute to autophagosome formation [66]. In mammalian cells, ER-isolation membrane (IM, precursor of the autophagosome) is modulated by VAPs interacting with multiple ATG proteins, contributing to autophagosome formation. Furthermore, ATG2 has been reported to mediate the ER-autophagosome MCSs (EACSs) with ATG18, serving as a lipid-transfer protein in forming autophagosomes [65]. The Arabidopsis ORP2A (oxysterol-biding protein related protein 2A) forms a complex with VAP27-1 and ATG8e to mediate EACSs upon autophagic induction (Figure 2B). In ORP2A knock down (KD) plants, autophagic proteins and PI3P accumulated on the ER membrane, while autophagosome formation is impaired, indicating that OPR2A may serve as a PI3P regulator in autophagosome initiation [67]. These findings highlight the roles of MCSs in regulating plant autophagosome formation and organelle communication in plants.

### Crosstalk between ATG9 vesicles and distinct plant SNAREs in autophagosome formation

In yeast and mammals, specific SNAREs are required for the homotypic fusion of ATG9 vesicles and phagophore precursors for autophagosome biogenesis [68,69]. In plants, only one Arabidopsis SNARE, VTI12 (vesicle transport v-SNARE 12), was shown to play a potential role in mediating autophagosome-vacuole fusion [70]. In Arabidopsis, depletion of ATG9 affects autophagic flux where abnormal tubular autophagosome-ER structure was observed in the *atg9* mutant [71]. Cryo-EM structures of the ATG9 revealed that ATG9 oligomerization is crucial and may promote ATG9 vesicle budding to the PAS [72].

Two plant SNARE proteins, VAMP724 and VAMP726, were recently shown to regulate autophagosome formation as they colocalize with the autophagosome marker ATG8e and may function together with ATG9 vesicles [73] (Figure 2C). Interestingly, similar tubular autophagosome-ER structures found in the *atg9* mutants [71] were also found in the *vamp724 vamp726* double mutants, indicating these two SNARE proteins and ATG9 vesicles likely function in the same pathway [73]. Indeed, the distribution of ATG9 puncta was affected in the *vamp* mutants while the subcellular localization of VAMP724 and VAMP726 remained normal in the *atg9* mutant, supporting the notion that VAMP724 and VAMP726 regulate ATG9 trafficking to contribute to autophagosome biogenesis.

### Distinct COPII populations contribute to phagophore initiation and expansion

Conventionally, COPII vesicles transport proteins and lipids from the ER to the Golgi apparatus (Figure 2, top panel), while they also play roles in autophagosome biogenesis. Studies in yeast showed that the phosphorylation of COPII cargo adaptor SEC24 reprograms COPII vesicles to fuse with the phagophore by interacting with ATG9 [74]. In mammalian cells, COPII vesicles that are specifically derived from ER–Golgi intermediate compartment (ERGIC) contain SEC23B protein and contribute as a membrane source to the phagophore precursor [75,76]. Plants harbor functionally diverse COPII components, including multiple homologs of SAR1 (secretion associated RAS 1), SEC23, SEC24, SEC13, and SEC31 [77]. Those plant-unique homologs play multiple functions in plants, including the SAR1a-mediated giant COPII vesicle formation enriched with membrane transporters in response to drought stresses [77], pollen development [78], and autophagy [79,80] in Arabidopsis.

SAR1D, a plant-specific SAR1 homolog, was found to interact with RABD2a (a plant-unique RAB1/YPT1 homolog) and the ATG machinery to regulate a specific COPII population in autophagy [81] (Figure 2D). The RABD2a-AtSAR1D nexus serves as a molecular switch to redirect COPII vesicles from the canonical pathway to the autophagosome biogenesis pathway, contributing to autophagosome initiation as a membrane source. Interestingly, SAR1D, but not other SAR1 paralogs, specifically regulates this autophagosome biogenesis process. In addition to SAR1D, a large-scale proteomic analysis has identified several other COPII components such as Sec24-like CEF and SEC23f involved in the process [81]. SEC23 homologs function diversely in the formation of ER exit sites [82], yet which SEC23 or SEC24 homolog functions in autophagy-related COPII population remains elusive. The COPII machinery is also involved in autophagosome maturation steps. The interaction between FYVE2 and the COPII component SAR1B in autophagosome elongation was revealed recently [80] (Figure 2E). Taken together, functionally diverse COPII components mediate distinct pathways and developmental stages in specific environments in plants.

### Crosstalk between autophagosome maturation and endocytic pathway

The ESCRT complex is involved in the endocytic pathway, mediating endosome maturation and cargo degradation (Figure 2, top panel). In both mammalian cells and yeast, ESCRT components have been shown to contribute to autophagosome closure [83,84]. In plants, several ESCRTIII components such as CHMP1 (charged multivesicular body protein 1) [85] and AMSH3 (associated molecule with the SH3 domain of STAM3) [86] were previously shown to play a role in autophagosome closure.

The identification of the plant unique ESCRT component FREE1 revealed direct crosstalk between autophagosome biogenesis and endocytic pathway. FREE1 is a unique plant ESCRT component that regulates multivesicular body (MVB) biogenesis and vacuolar protein transport as well as autophagosome degradation [87]. The Arabidopsis *free1* mutant showed defects in both intralumenal vesicle (ILV) formation in MVB and central vacuole formation, as well as accumulations of autophagosomes and lipid droplets [88,89]. Further investigations showed that FREE1 directly participated in the autophagy pathway via interacting with autophagosome membrane-located autophagy regulator SH3P2 (SH3 domain-containing protein 2) [90].

Recently, Zeng et al. (2023) [79] investigated how FREE1 shuttles between the traditional endocytic pathways and autophagosomes (Figure 2F). Under non-stress condition, FREE1 works together with other ESCRT components to regulate endosomal sorting and MVB biogenesis. During starvation, SnRK1 (KIN10) suppresses the TOR signaling pathway and phosphorylates FREE1, which interacts with ATG8 and the ATG12-ATG5-ATG16 conjugation system, to recruit ESCRTIII components to regulate autophagosome closure. KIN10 serves as a switch turning FREE1 into a new role via its phosphorylation status. Indeed, both FREE1 protein *per se* and its phosphorylation process are necessary for autophagosome closure, as the *free1* mutant and FREE1^S530^^AS533A^/*free1-ct* phosphorylation-deficient mutant showed accumulation of “unclosed” autophagosomes [79].

### Role of autophagosome-MVB fusion in amphisome formation

The process of autophagosome biogenesis and maturation varies among yeast, metazoans, and plants. In yeast, autophagosomes form and mature near the vacuole, benefiting from their close spatial relationship [91]. In contrast, metazoans generate autophagosomes at diverse cellular locations, prior to their fusion with endosomes/lysosomes to produce amphisomes [92]. In plants, autophagosomes distribute throughout the cell and are destined to the central vacuole [93]. However, it remains elusive whether plant autophagosomes undergo amphisome formation before reaching the central vacuole. Recently, the role of CFS1 (cell death related endosomal FYVE/SYLF protein 1) as an autophagy adaptor for amphisome formation in plants has been investigated [94] (Figure 2G). CFS1 interacted with both ATG8 via AIM and VPS23A (vacuolar protein sorting 23A), a MVB-localized ESCRT-I complex component, playing a bridging role in recruiting autophagosome and MVB together for the formation of an amphisome [94] (Figure 2G).

In summary, recent studies have shed new lights on the crosstalk and participation of the endomembrane trafficking in autophagosome biogenesis with distinct mechanisms in plants. However, several outstanding questions are waiting for answers in future studies: (i) In addition to the known ER- and PM-autophagosome connections, is there other membrane contact site playing role in regulating autophagosome biogenesis? (ii) The plant unique ESCRT component FREE1 plays multiple roles in regulating membrane trafficking and organelle biogenesis in both endomembrane system and autophagic pathway with distinct mechanisms in plants, are there other plant unique components playing multiple roles? (iii) What are the spatio-temporal resolutions in regulating the precise steps of autophagosome biogenesis from phagophore growth to autophagosome closure?

## Autophagy meets receptor biology

Eukaryotic cells have evolved receptor proteins to sense and respond to environmental cues and intrinsic demands. In plant biology, receptor proteins are heavily associated with developmental or immune responses, where, for example, cell surface localized receptors sense damage associated or developmentally regulated small peptides to induce an immune or developmental signaling cascade [95]. The common feature of these receptors is that they are specific to certain stimuli and trigger an adaptive signaling response [96]. Studies in the last decade have shown that autophagy is selective, and this selectivity emerges from dedicated receptor proteins known as selective autophagy receptors or cargo receptors [97-100]. Cargo receptors are modular proteins. They (i) have cargo binding or recognition domains, which confer selectivity, and (ii) interact with ATG8 and other core autophagy proteins to bridge the cargo with the growing autophagosomes [101,102]. Similar to the cell surface receptors, depending on the cargo and the nature of the selective autophagy response induced under stress or developmental transitions, each cargo receptor is connected to a distinct homeostatic response that works harmoniously with the rest of the cellular signaling and metabolism [103,104]. Therefore, identification and characterization of cargo receptors are key to understand the role of autophagy in plant homeostasis [105].

Mechanistic details of how cargo receptors recruit cargo to the autophagosomes mostly come from studies in human cells. Even though cargo receptor studies are still in their infancy in plants, several studies in the last few years have discovered new cargo receptors that mediate protein or organelle degradation. Some of these mechanisms turned out to be also conserved in mammalian cells or yeast, illustrating the power of plants as organismal model systems to study autophagy mediated cellular quality control.

### Plant cargo receptors that degrade proteins and protein aggregates

The best studied cargo receptor in plants is NBR1 (neighbor of brca1), which is a hybrid form of mammalian p62 and NBR1 proteins [106,107]. The Arabidopsis NBR1 can form oligomeric structures via the PB1 domain and interact with ubiquitinated aggregates and ATG8 via to the ubiquitin-associated (UBA) domains and the AIM, respectively [106,107]. Whether it also interacts with FIP200 homolog ATG11 or cooperates with other hitherto unknown receptors is unknown. Functionally, NBR1 is crucial for clearing protein aggregates that arise during abiotic or biotic stress conditions [108-113]. Although the specific cargo degraded by NBR1 during stress conditions haven’t been catalogued in depth, NBR1 was shown to degrade small heat shock proteins to regulate heat stress memory [114]. Further studies are necessary to understand NBR1-mediated stress tolerance mechanisms.

**Figure 3.**
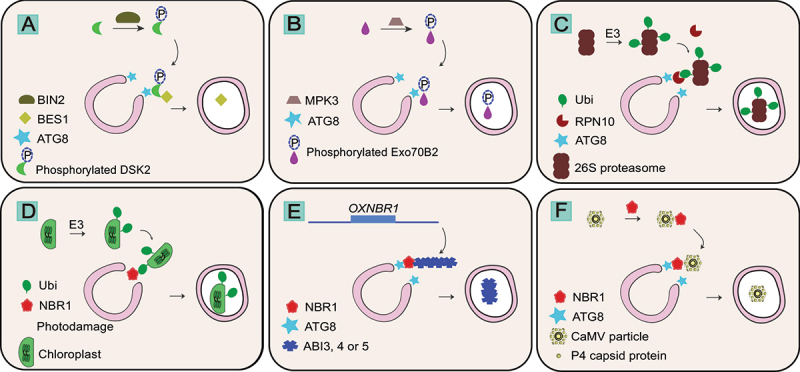
Examples of selective autophagy targeting proteins, organelles, and pathogens in plants. (A) During drought or carbon starvation BIN2 phosphorylates DSK2 which targets BES1 to ATG8 for subsequent autophagic degradation. (B) Upon immune activation MPK3 phosphorylates Exo70B2 which binds to ATG8. (C) Ubiquitinated proteasome binds RPN10 under proteotoxic stress or nitrogen starvation leading to proteophagy. (D) Photodamaged chloroplast gets ubiquitinated leading to chlorophagy. (E) NBR1 targets ABI transcription factors for autophagic degradation when overexpressed. (F) NBR1 targets the capsid protein P4 and entire viral particle leading to xenophagy of cauliflower mosaic virus (CaMV). See text for details.

In addition to NBR1, another UBA domain containing protein, DSK2 was also shown to function as a cargo receptor. DSK2 degrades a key regulator of brassinosteroid signaling, BES1 (BRI1-EMS suppressor 1), upon drought stress or carbon starvation [115]. BIN2 (brassinosteroid insensitive 2), another key regulator of brassinosteroid hormone signaling, phosphorylates DSK2 residues around the AIM, strengthening ATG8 interaction and leading to the activation of DSK2 cargo receptor activity [115]. Activated DSK2 could in turn recruit BES1 into autophagosomes for degradation [115] (Figure 3A). Since BES1 is also degraded via the proteasome, it could serve as a model substrate to dissect how plant cells decide to employ autophagy over the proteasome [116].

Another cargo receptor that is regulated by phosphorylation is the exocyst subunit EXO70B2 [117]. Upon elicitor or salicylic acid analog BTH treatment, EXO70B2 gets phosphorylated by MPK3 (mitogen-activated protein [MAP] kinase 3) around the AIM residues [117]. This increases ATG8 affinity and activates autophagic degradation of EXO70B2 [117] (Figure 3B). Since EXO70B2 normally localizes at sites of active secretion such as the tip region of root hairs, autophagic degradation is proposed to dampen exocyst mediated secretion [117]. Although some other exocyst subunits such as EXO70A1 or SEC6 also undergo vacuolar degradation, whether their degradation is mediated by EXO70B2 remains unknown. Also, if EXO70B2 mediates the degradation of other proteins is not studied so far. However, another EXO70 isoform, EXO70D1-3 has been shown to function as a cargo receptor that selectively degrades type A response regulators in Arabidopsis [118]. Type-A ARRs (*Arabidopsis thaliana* response regulators) are negative regulators of cytokinin signaling [119]. They are also well-known proteasome substrates [119]. Upon phosphorylation, they interact with EXO70D and become an autophagy substrate to regulate cytokinin outputs [118].

In addition to phosphorylation, ATG8-cargo receptor interaction is also regulated by S-nitrosylation in Arabidopsis [120]. During hypoxia, GSNOR (S-nitroglutathione reductase), a key regulator of nitric oxide signaling gets S-nitrosylated. This leads to a conformational change, where a normally buried AIM becomes accessible for ATG8 interaction and autophagic degradation [120]. Whether GSNOR also mediates the degradation of other proteins remains unknown.

These three examples highlight how plants employ selective autophagy to regulate various signaling and trafficking mechanisms. In contrast to the conceptual model that we described above, how the cargo forms bulky aggregates to become autophagy substrates and how other members of the core autophagy machinery except ATG8 are brought to the aggregation site need further investigation.

Nevertheless, these examples highlight the close crosstalk between the proteasome and the autophagy pathway. Accumulating evidence suggests that aggregates are initially channelled to the proteasomes, since it is rapid and does not involve the biogenesis of a vesicle. Once these aggregates become too bulky to be handled by the proteasome, autophagy is employed to clear them out [121]. Autophagy-proteasome crosstalk is not limited to the substrates. Upon proteotoxic stress or nitrogen starvation, the 26S proteasome has been shown to be degraded via autophagy in Arabidopsis, known as proteaphagy [122]. The ubiquitin receptor RPN10 (regulatory particle non-ATPase 10) can bind ATG8 to recruit proteasomes to the autophagosome [122] (Figure 3C). Similarly, another ubiquitin binding protein family, PUX (plant UBX domain-containing protein) was shown to recruit CDC48 (cell devision cycle 48) complexes to the autophagosomes [123]. Disease associated mutations increased the autophagic flux of CDC48, suggesting autophagy clears out non-functional CDC48 complexes to prevent cytotoxicity [123].

Interestingly, both RPN10 and PUX proteins contain ubiquitin interacting motifs (UIMs) and were suggested to interact with ATG8 via a non-canonical site, defined as ubiquitin docking site (UDS) [123]. UDS is in close proximity to the ATG8 C-terminus that gets lipidated to attach to the phagophore [123]. How receptors could fit into this region without disrupting membrane interaction remained puzzling. Furthermore, in solution NMR studies of yeast ATG8 have shown that the region defined as UDS interacts with the phagophore membrane to cause membrane bending, which is crucial for autophagosome biogenesis [124]. Therefore, further studies are necessary to elucidate how proteasome and CDC48 complexes are degraded via selective autophagy and how UIM-type cargo receptors could function without disrupting ATG8 function.

Another UIM-containing protein was discovered from virus infected Arabidopsis plants. A 71 residues long peptide, VISP1 (virus induced small peptide 1), was identified as a viral induced small open reading frame. Overexpression of VISP1 induced autophagy and triggered the degradation of phase separated RNA-protein granules known as SGS3 (suppressor of gene silencing 3)-RDR6 (RNA-dependent RNA polymerase 6) bodies [125]. Whether VISP1 could interact with UDS due its small size or there are additional adaptors involved in VISP1 induced autophagy remains unknown. In addition to viral infection, SGS3 bodies are also induced and degraded via autophagy upon hypoxia, and this degradation requires CML38 (calmodulin-like protein 38) [126]. However, it is not clear if CML38 functions as a cargo receptor or triggers SGS3 autophagic degradation in another way.

### Organelle recycling via selective cargo receptors

In contrast to soluble proteins, we know less about selective autophagy of organelles. There is accumulating evidence suggesting that, similar to yeast and metazoans, organelles are degraded via autophagy in plants, but the cargo receptors that mediate organelle recycling and particularly their mechanism of action is far less understood.

For endoplasmic reticulum, similar to mammalian cells, reticulon proteins were shown to mediate ER-recycling in maize. Particularly, in maize endosperm Rtn1 (reticulon 1) and Rtn2 proteins remodel the endoplasmic reticulum to maintain homeostasis [127]. Another conserved ER-phagy receptor is SEC62. It was initially discovered as an cargo receptor in human cells that removes excess ER formed during stress [128]. Following studies in Arabidopsis have shown that the recoverophagy function of SEC62 is also conserved in plants [129]. Finally, C53 was initially discovered in plants to function as an ER-phagy cargo receptor that is activated upon stalling of ER-bound ribosomes [130]. Further studies have shown that C53 also functions as an cargo receptor in human cells and its activity is regulated by UFM1 (ubiquitin fold modifier 1) [130,131]. Another family of cargo receptors is the ATI (ATG8 interacting protein) family proteins [132]. They have transmembrane domains and localize to ER and plastid associated bodies [132-134]. Recent studies have shown that ATI family could mediate the degradation of ER-associated AGO1 (argonaute 1) protein and ER-inserted MSBP1 (membrane steroid binding protein 1), during viral infection and starvation, respectively [133,134]. How these receptors remodel the ER, fragment it and recruit the cargo into autophagosomes remains for further investigation.

Besides ER-recycling, there is a growing interest in chloroplast and mitochondria autophagic recycling in plants. There are several reports demonstrating chloroplast degradation via macro- or microautophagy pathways [135]. Recent studies have shown that NBR1 plays a role in degradation of chloroplasts, plausibly upon loss of chloroplast integrity [112,136] (Figure 3D). However, whether plants have evolved dedicated chlorophagy receptors remain unknown. For mitochondria, it was shown that FMT (friendly), a REC-family protein involved in the regulation of mitochondrial abundance and division, is essential for mitophagy [137-139]. In *fmt* mutants, mitochondria aggregate and uncoupler induced mitophagy is prevented [137-139]. Excitingly, FMT-mediated mitophagy was also crucial for de-etiolation, a major cellular reprogramming response that involves the maturation of chloroplasts from etioplasts [137]. Whether mitophagy is crucial for deetiolation due to the energy demand required for reprogramming, or mitochondria and chloroplasts form interconnected networks that will require both organelles to be reprogrammed during deetiolation are exciting hypotheses that need to be tested. In addition, other members of the REC family regulate chloroplast distribution and abundance [140]. Whether they are involved in chlorophagy remains unknown. In addition to FMT, TRAB proteins were recently shown to regulate mitophagy [141]. TRAB proteins localize to the ER-mitochondria contact sites, where they colocalize with the known ER-plasma membrane contact site protein VAP27-1 [141]. Similar to FMT, TRAB proteins regulate mitochondrial abundance and morphology. Furthermore, they were shown to interact with ATG8 via an AIM and regulate uncoupler induced mitophagy [141]. Previous studies have shown that VAP27 proteins also regulate endocytosis associated autophagy, cytoskeletal dynamics, and lipid trafficking [66]. As such they form a dynamic signaling hub where biogenesis, energy production, and degradation mechanisms could crosstalk to maintain cellular homeostasis, despite changing environmental conditions and intrinsic demands.

In summary, despite the growing list of selective cargo receptors in plants, their molecular mechanism of action, cargo, and crosstalk with other homeostatic pathways remain largely unknown. Since the cargo is playing an active role in activating selective autophagy, cargo receptor discovery approaches should focus on comparative analysis of physiological stress conditions that would trigger different selective autophagy responses. Also, the cargo receptor catalogue will differ in each cell type. Developmentally regulated mitophagy that happens during sperm maturation in mitochondria may not be similar to the mitophagy pathways triggered during de-etiolation [142]. Nevertheless, comparative analyses of these responses will reveal the extent to which plants employ cargo receptors to shape and remodel their cytoplasm.

## Autophagy and development

Since the discovery of the first autophagy-deficient plant mutants in Arabidopsis, autophagy has, in plants as well as in animals, been considered as a pro-longevity process. While autophagy deficient plants thus exhibit early onset of chlorosis, they develop remarkedly normal at the macroscopic level under controlled growth conditions [44,143]. Nevertheless, autophagy affects most, if not all aspect of plant development including seed, vasculature tissue, root, and reproductive development [144,145]. In line with this, plant hormones are also implicated in the regulation of autophagy and strongly affected by autophagy [146,147]. A well-studied example includes the plant growth hormone brassinosteroid (BR) that may both regulate autophagy [148] and is regulated by autophagy [115,149].

Thus, as testified by these and numerous other reviews and reports, we are digging in on autophagy in development. A recent example includes the study of root cap development in Arabidopsis. The root cap is kept at a constant size by cell death and shedding and autophagic activity increase in pre-programmed cell death (PCD) of lateral root cap cells [150,151]. To study more directly the role of autophagy in developmental PCD in the root cap, Feng and colleagues generated transgenic lines with root cap specific KOs of *ATG2* or *ATG5*. Sloughed root cap cells in both complete *atg5* and cell type specific *atg2* and *atg5* KOs remained viable significantly longer than in wild type [150], thus directly demonstrating an autonomous function of autophagy in the developmental PCD required to form normal root caps.

In addition to studying autophagy deficient plants, Wang and colleagues recently reported increased autophagic activity in Arabidopsis *feronia* mutants [152]. *FERONIA* (*FER*) encodes a receptor-like kinase which functions together with co-receptors to regulate many aspects of plant development including pollen tube growth, optimal vegetative growth, and root hair development [153]. Autophagic activity is markedly increased in *fer* mutants while TOR kinase activity is severely compromised, and this is also true when overexpressing TOR in *fer* mutants. Oppositely, overexpression of *FER* is sufficient to inhibit autophagy induction by sucrose starvation but only in wild type plants, not in *raptor1b* mutants placing FER directly upstream of TOR kinase function [152].

The GSK3 (glycogen synthase kinase 3)-like kinase BIN2 functions as a negative regulator of BR responses. Thus, in the presence of BR, BIN2 is kept inactive to favor growth. However, in contrast to FER, BIN2 was recently shown to positively regulate autophagy. More precisely, BIN2 can both be found in proximity and to directly phosphorylate RAPTOR1B [148]. RAPTOR1B functions to recruit substrates to TOR and Liao and colleagues [148] found that when BIN2 is inhibited by BR, RAPTOR1B phosphorylation is decreased. This promotes phosphorylation of ATG13a by TOR and inhibition of autophagy. Thus, when BR is absent, BIN2 can phosphorylate and suppress the TOR complex through RAPTOR1B to induce autophagy. With these two examples the authors speculate that FER and BIN2 are involved in regulating TOR function to balance nutrient and energy levels via autophagy. Nevertheless, it is still unknown how directly or indirectly FER is linked to autophagy.

Recent evidence also points to the involvement of autophagy in seed development in Arabidopsis since autophagy deficient plants exhibit a variation of abnormality of embryo development [154]. In support of this, *ATG8* transcripts accumulate in mature green embryo-bearing siliques compared to earlier developmental stages and autophagic activity also increases in the developing siliques [154]. *ATG* gene expression has also recently been reported to increase during fruit ripening in strawberry, and tomato *ATG4* RNAi lines showed reduced fruit growth, supporting the notion that autophagy contributes to fruit development [155,156].

To study how autophagy in the maternal tissue may affect seed development Erlichman and co-workers [157] elegantly performed reciprocal crosses between *atg* mutants and wild type plants. These crosses revealed that F1 seedlings derived from an autophagy-deficient mother exhibited shorter hypocotyls than seedlings originating from a wild type mother. Since the protein content was also significantly reduced in F1 seeds originating from autophagy deficient mothers, the authors concluded that autophagy is involved in the regulation of carbon and nitrogen allocation from the mother plant to the seed [157]. Importantly, the authors could demonstrate that the shorter hypocotyl was independent of the senescence associated phenotype seen in *atg* mutants.

Recent findings also indicate that autophagy modulates lateral root (LR) formation. Ebstrup and co-workers found that *atg* mutants display reduced numbers of LRs and that NBR1 mediates the selective autophagic degradation of AUXIN RESPONSIVE FACTOR 7 (ARF7), a key regulator of LR formation [158]. The authors further showed that auxin promotes the co-localsation of ARF7 with NBR1 and ATG8a leading to its autophagic turnover. Concordantly, when autophagy was impaired either chemically or genetically, ARF7 accumulated in nuclei and cytoplasmic condensates, causing decreased auxin responsiveness and consequent defects in LR pre-positioning and formation.

However, a major obstacle in studying autophagy deficient mutants can be the pleiotropic and secondary effects of knocking out such a central degradation system at the organismal level. Rodrigues and colleagues [9], for example, recently demonstrated that practically all plant hormones, microbial and danger associated molecular patterns induced a rapid activation of autophagy. Thus, autophagic deficient plants accumulate vast amounts of proteins representing signatures of earlier endogenous and exogenous signalling events which they are unable to degrade. Consequently, autophagic deficient mutants first struggle to make cellular changes exemplified by reduced and delayed callus formation. However, at later stages the responses may become exaggerated as illustrated by bulky callus development when explants are placed on shoot inducing media [9]. Oppositely, plants overexpressing autophagy are simply better at adapting and make cellular changes, probably because they rapidly degrade signatures from previous cellular programs which smoothen transitions [159,160].

In summary, and as also mentioned above, much of what we have learned about autophagy in development is based upon observations done in autophagy-deficient plants. However, it cannot be surprising that loss of autophagic activity from fertilization can mask the role of this process in plant development. Autophagy itself may thus not directly regulate plant development. The “blurred proteome” in autophagic deficient plants can have a negative impact on plant development. Its role in maintaining proteostasis is essential for ensuring that cells function properly during multiple developmental transitions. Thus, tissue-specific knockout mutants as for example the ones used by Feng and co-workers [150] may help further clarify the developmental consequences of autophagic deficiencies, maybe in combination with conditional KOs.

## Autophagy and metabolism

While autophagy delivers macromolecules and even entire organelles to a lytic organelle [161], the interaction of autophagy and metabolism displays considerable differences with that observed in yeast and mammals. These are due both to inherent differences in the metabolism of the species as well as the different requirements each organism requires to maintain optimal fitness [162]. Mega-autophagy, in which the disintegration of the tonoplast results in the release of a suite of enzymes including proteases and hydrolases into the cytosol, results in the degradation of cytosolic material and eventually cell death [163,164]. It has also been characterized to be important during the formation of secondary xylem in poplar [165]. That said both the interaction of megaautophagy and microautophagy with metabolism are relatively poorly studied so we will concentrate this section solely on the interaction of macroautophagy (hereafter simply referred to as autophagy) and metabolism. Moreover, given that their roles have been extensively reviewed elsewhere [166–168] we will not cover the roles of TOR or SnRK1 in this interaction.

An obvious place to start discussing the interaction of autophagy and metabolism is at the level of transcription. While transcriptional regulation of the core machinery is described above its role in the interface of autophagy and metabolism is considerable. Indeed, the majority of ATG8 genes in both Arabidopsis and soybean (*Glycine max*) exhibits rapid increases in gene expression following nitrogen deprivation [169,170], yet by contrast only a small number of the ATG8 genes are enhanced in expression following several days of carbon starvation [169,171,172]. Beyond ATG8 several other of the ATG genes are induced on nutrient stress with ATG18 being responsive to both carbon and nitrogen starvation [173] and several ATG genes being induced by prolonged darkness in *N. benthamiana* [174]. Although the transcription factors responsible for regulating *ATG* gene expression are well characterized in yeast and mammalian cells [175], in plants knowledge is currently restricted to that concerning HSFA1A, ERF5 (ethylene responsive element binding factor 5), BZR1 (brassinazole resistant 1) and WRKY 33 and 45 [20,169,176-178] as well as the transcriptional regulator HY5 [179]. However, despite the identification of these transcription factors alongside the detailed characterization of the expression of the autophagy genes themselves neither the gene-regulatory networks nor more importantly their metabolic triggers are yet fully understood in plants and their study remains an important priority.

The importance of autophagy both under nutrient-rich and -deficient conditions has been well documented for multiple plant species. Under nutrient-rich conditions Arabidopsis and maize autophagy mutants display considerable metabolic changes with a compromised nitrogen remobilization with increased protein levels and altered lipid metabolism being a characteristic of both species, while amino acids and ammonia were elevated only in Arabidopsis [180-183]. Similarly, detailed characterization of tomato plants that were deficient in the autophagy-regulating protease *ATG4* revealed that these displayed an early senescence phenotype yet relatively mild changes in the foliar metabolome when grown under nutrient-rich conditions [156]. Indeed, the levels of many fruit primary metabolites exhibited decreases in the *ATG4*-RNAi lines, such as proline, tryptophan and phenylalanine, whilst representative secondary metabolites were present at substantially higher levels in *ATG4*-RNAi green fruits than in wild type. Furthermore, integration analysis of the metabolome, transcriptome and proteome data indicated that ATG4 significantly affected lipid metabolism, chlorophyll binding proteins and chloroplast function [156].

A common plant stress is the lack of carbon availability caused by insufficient light irradiance. Autophagy is one of three mechanisms which promotes the degradation of carbon sinks, the other two processes being proteasome-mediated degradation and chloroplast vesiculation. Autophagy has for example been suggested to contribute to transitory starch degradation in Arabidopsis [174] and been found to be induced by C starvation in several other species. For instance, *ATG* transcripts and ATG-PE conjugates being elevated in maize on C stress [184], whilst overexpression of ATG8 in apple lead to an increased tolerance to C stress [185]. Similarly, metabolic profiling of etiolated Arabidopsis *atg* mutants displayed reduced levels of amino acids and elevated protein contents [186], with a similar phenotype observed when mature plants were exposed to continuous darkness [187] and in *atg* mutants grown under short-day conditions [188]. In maize *atg* mutants the changes described above in nutrient rich conditions were exacerbated with major increases in sugar, organic and amino acid levels as well as in starch degradation [183]. Such effects of autophagy are not, however, confined to the core central metabolism with a clear role for the process emerging in lipid homeostasis [161]. Indeed, this was observed both in the etiolated Arabidopsis seedling experiment described above [186] and has been the subject of a recent comprehensive review which we refer the reader to [189]. It is worth mentioned that the changes observed in both central metabolites and lipids are not all conserved across species with considerable differences being observed between for example Arabidopsis [190] and maize [183] suggesting that it will be highly important to expand the range of such studies in the future to accommodate a greater number of species and tissue types in order to better understand the different metabolic contexts in which autophagy operates.

The picture is further muddied by recent findings of the significance of S and P stress in the interaction between autophagy and metabolism. Given that considerably less research has focused on these nutrients and they have been extensively reviewed recently [161], we will only discuss them briefly together here. Considerable further research is needed to understand the interaction of both S and P and autophagy. Sulfide induced persulfidation of specific cysteine residues on ATG4 and ATG18a [45,191,192], however, this effect is independent of the role of S as a nutrient since it also occurs under S sufficiency [193]. The central metabolite which coordinates S, C and N flux in plants – cysteine – has also been demonstrated to play an important role in Glucose-TOR signaling [194] which has considerable overlap with autophagy. Similarly, P starvation induces autophagy in several species causing ER stress in for example tobacco [195,196] while Arabidopsis mutants are hypersensitive to P limitation [197]. Many studies have centered on the role of autophagy in nitrogen (N) mobilization in several crop species and will be discussed in detail in the section “*Autophagy in crops”* below.

Although we have learned a lot about the extensive interaction between metabolism and autophagy many open questions remain. While the study of constitutive mutants and transgenics have largely supplied the above knowledge, future experiments that shift towards inducible manipulations of autophagy and/or metabolism will likely issue in a more detailed understanding of this fascinating yet highly complex interplay. The very recent findings that three consecutive enzymes of glycolysis and FCS-like zinc finger (FLZ) proteins regulate autophagic flux [198,199] is an intriguing novel aspect at the interface of autophagy and metabolism, and how metabolism influences autophagy represents a key future research front.

## Autophagy and abiotic stress

One of the earliest phenotypes described for Arabidopsis *atg* mutants is hypersensitivity to abiotic stresses, such as heat, drought (osmotic), salt, ER, and oxidative stress [200]. In addition, overexpression of *ATG* genes in Arabidopsis resulted in increased resistance to oxidative stress [159]. These data indicate that an increased understanding of the role of autophagy in abiotic stress resistance might aid in developing novel agricultural solutions, specifically in light of the current climate change. Several excellent reviews have been published in recent years describing the roles of autophagy in abiotic stress and its agricultural importance [201]. That said, common themes have emerged from manuscripts published in the last few years. This section will describe them.

The study of autophagy in plants has expanded to many plant species in recent years, including wild plants, specifically regarding abiotic stress response. Autophagosomes were shown to accumulate in shoots of the resurrection plant *Tripogon loliiformis* during desiccation. Autophagy induction was mediated by trehalose accumulation [202], suggesting a possible role for regulatory sugars in autophagy induction. Interestingly, trehalose accumulation was also observed in *Paspalum vaginatum*, a wild relative of maize and sorghum, which is resistant to many abiotic stresses. Inhibiting trehalose breakdown in maize resulted in increased biomass in an autophagy-dependent manner [203]. Other works have highlighted autophagy as a survival strategy for plants acclimated to harsh environments. Samples of the shrub *Caragana korshinskii* collected from locations with various drought conditions demonstrated the existence of autophagosomes under drought conditions as well as increased expression of *ATG* genes. The authors correlated this expression with sugar concentrations in the plants. However, a direct connection has not been established [204]. In addition, increased expression of *ATG3, ATG4, ATG7* and *ATG8* was observed in leaves of the halophyte *Eutrema salsugineum* under salt stress [205].

**Figure 4.**
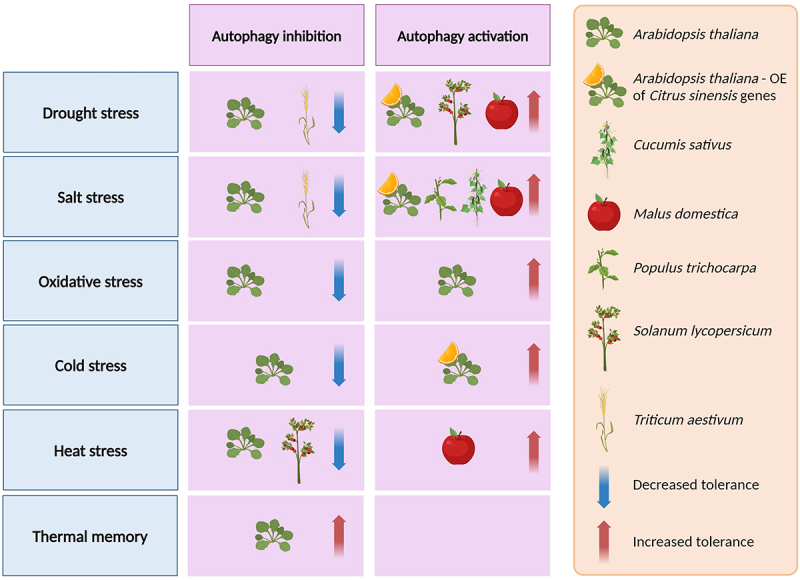
The contribution of autophagy to abiotic stress tolerance in plants. The figure summarizes the observed effects of altered autophagy levels on abiotic stress tolerance in different plant species. Autophagy inhibition was achieved by loss-of-function mutations in *ATG* genes and the *NBR1* cargo receptor or by chemical means. Autophagy activation was mediated by overexpression (OE) of *ATG, NBR1* and other genes, as well as loss-of-function mutations in negative autophagy regulators or chemical treatment. See text for details. The figure was created with BioRender.com.

Studies from Arabidopsis have shown that *atg* mutants are hypersensitive to abiotic stress. This phenotype has been expanded to additional plant species, specifically crops (Figure 4). Down-regulation of *ATG* genes in tomato (*Solanum lycopersicum*) resulted in increased sensitivity to heat stress [20]. In addition, inhibition of autophagy in wheat (*Triticum aestivum*) seedlings increased their sensitivity to drought and salt stress [206,207]. Moreover, as opposed to testing the phenotype of whole plants under stress conditions, the effect of autophagy at the tissue level was also investigated. Arabidopsis *atg* mutants exposed to heat stress displayed aberrant pollen development and increased male sterility [208]. Alternatively, examples from various plant species demonstrate improved abiotic stress tolerance due to the overexpression of *ATG* genes. As mentioned above, overexpression of *AtATG5* and *AtATG7* in Arabidopsis resulted in increased resistance to oxidative stress [159]. Similar results were observed in additional plant species, such as apple (*Malus domestica*) and sweet orange (*Citrus sinensis*), in response to a variety of abiotic stresses, such as drought, heat, salt, and cold stress. Improved phenotypes were observed when *ATG* genes were overexpressed in the same species or in Arabidopsis [209-213] (Figure 4).

More insight was gained regarding the mechanisms behind autophagy induction during abiotic stress. Accumulating evidence from past and recent studies point to the involvement of reactive oxygen species (ROS), such as H_2_O_2_. Research from the model alga *Chlamydomonas reinhardtii* revealed that autophagy induction during ER stress results from oxidative stress. Addition of the antioxidant glutathione partially suppressed ER stress-induced autophagy [214]. In addition, metabolic analysis of developing maize seeds revealed that *atg* mutants hyper-accumulated metabolites associated with oxidative stress, suggesting this stress is alleviated by autophagy [215].

The source of ROS is still under debate. ROS were shown to mediate autophagy induction by spermidine in cucumber (*Cucumis sativus*) exposed to salt stress. Treatment with spermidine induced the expression of *ATG* genes and increased plant stress resistance [216]. In Arabidopsis, an alkaline ceramidase (*AtACER*) possibly functions in ROS-dependent autophagy induction [217]. Research in tomato revealed that alternative oxidase (AOX) induces autophagy during ethylene-dependent drought response. Plants overexpressing AOX1a displayed enhanced resistance to drought stress and increased autophagosome numbers upon treatment with the ethylene precursor ACC (1-aminocyclopropane-1-carboxylate). The authors attributed this increase to ROS accumulation, followed by ethylene-dependent expression of *SlATG8d* and *SlATG18h* [218]. Interestingly, quantitative trait loci (QTL) analysis revealed that *ATG18* also functions in drought stress in maize (*Zea mays, ZmATG18b*) [219] and rice (*Oryza sativa, OsATG18a*) [220]. Autophagy is also implicated in ROS scavenging. Overexpression of *MdATG8a* in apple led to reduced ROS accumulation upon heat stress [211]. This dual function was also demonstrated in wheat plants exposed to waterlogging. On the one hand, *ATG* gene expression was induced by waterlogging and ROS treatment. On the other hand, chemical induction or inhibition of autophagy led to increased or decreased ROS clearance, respectively. This was more pronounced in a waterlogging-resistant cultivar [221].

Further contributing to our understanding on the regulation of autophagy, plant autophagy inhibitors have also been described in recent years. Hydrogen sulfide (H_2_S) negatively regulates autophagy induction [222]. Recent studies revealed that this inhibition is exerted by persulfidation of Cys residues of ATG proteins. This was shown for ATG4 in plants exposed to osmotic stress and ATG18a in plants exposed to ER stress [42]. During ER stress, the protein IRE1B functions in autophagy induction. The protein has both kinase and ribonuclease activities. An examination of both functions revealed that the endonuclease activity of IRE1B is necessary for autophagy induction, and this is presumably regulated by the transcript degradation of proteins that inhibit autophagy upon ER stress. Overexpressing several of the degradation targets of IRE1B resulted in autophagy inhibition upon ER stress [223]. COST1 was recently identified as a protein that inhibits autophagy under favorable conditions in Arabidopsis. *cost1* mutants display constitutive autophagy under normal conditions and increased drought tolerance. Interestingly, drought tolerance of *cost1* mutants is partially reliant on H_2_O_2_ signaling, further strengthening the connection between autophagy and ROS. The amounts of COST1 are negatively regulated by autophagy [54].

Selective autophagy has been implicated as the executor of abiotic stress response. Of note is the autophagy receptor NBR1. *nbr1* mutant plants are hypersensitive to drought and salt stress, demonstrating the accumulation of damaged proteins. Moreover, NBR1 overexpression in *Populus* resulted in increased salt tolerance [224]. Recent studies implicated NBR1 in the degradation of photodamaged chloroplasts either by microautophagy [112] or by macroautophagy of the translocon at the outer envelope membrane of chloroplasts (TOC) components, impacting chloroplast import [225]. In addition, novel selective autophagy receptors have been identified as abiotic stress modulators. One example is MtCAS31 (cold acclimation specific 31) from *Medicago truncatula*. The protein induces autophagy during drought stress via ATG8 binding to promote the autophagic degradation of the aquaporin MtPIP2;7 [226]. Other examples include the ER-phagy receptors, which alleviate ER stress caused by misfolded protein accumulation. These are C53, found in plant and animal systems, and the reticulon proteins Rtn1 and Rtn2 from maize [127,130]. In addition, the dicot-specific ATI3 was shown to bind UBAC2A and UBAC2B (ubiquitin-associated protein 2A/B) to facilitate heat and ER stress response [227].

Selective autophagy also modulates the transition from growth to survival through the regulation of plant hormones. NBR1 is presumed to modulate the ABA response to abiotic stress, as overexpression of NBR1 resulted in transcriptional changes of genes related to ABA signaling. The authors demonstrated binding of NBR1 to the ABI (ABA insensitive) 3, ABI4, and ABI5 transcription factors involved in ABA signaling [228] (Figure 3E). In Arabidopsis, the BR-related transcription factor BES1 is degraded by selective autophagy under starvation and drought stress. Surprisingly, autophagy was shown to be induced by BR during cold stress in tomato plants. The authors claim this is due to the transcriptional induction of *NBR1* and *ATG* genes by BZR1, another type of transcription factor functioning in BR signaling [229]. These findings raise the question whether the interplay between BR and autophagy is stress-specific. A recent publication revealed shared signaling elements between BR and TOR, highlighting the complexity of the interplay between hormonal and autophagy regulation [230].

An exciting emerging facet of studying autophagy and abiotic stress is the investigation of recovery from stress. NBR1 was shown to degrade proteins related to heat stress response during recovery from heat stress. Thus, the resulting phenotype of *nbr1* and *atg* mutants is increased thermal memory, stemming from the “lingering” of heat response proteins in the cell [114,231]. The ATI1 cargo receptor was also implicated in the degradation of proteins during recovery from heat stress and, thus, thermal memory regulation [232]. In addition, autophagy was shown to contribute to the production of dipeptides (pairs of amino acids stemming from the proteolytic activity and serving in regulatory roles) during recovery from heat stress [233]. Surprisingly, a recent publication demonstrated that ATG8 translocates to swollen Golgi membranes in an autophagy-independent manner to facilitate their recovery from heat stress, suggesting that autophagy components might serve independent functions during stress [234].

Another advance linking autophagy to field conditions is investigating its role in stress combinations rather than individual stresses. A recent publication revealed that combining high light and heat stress results in different physiological and metabolic responses than individual stresses. These differences were attributed to an accumulation of GABA (γ-aminobutyric acid), possibly inducing autophagy. Indeed, *atg* mutants and *gad3* mutants displayed hypersensitivity to the combined stress compared to control plants [156]. Another research investigated the interplay between drought stress and tomato yellow leaf curl virus (TYLCV) infection. Infection with TYLCV improved the drought tolerance of tomato plants. However, drought stress induced the expression of the transcription factor HSFA1, which was previously shown to promote autophagy induction by the transcriptional regulation of *ATG10* and *ATG18f* [19]. Expression of both genes was indeed enhanced following drought stress, and the authors postulate this increased autophagy promoted the degradation of TYLCV [235]. Further work is needed to uncover this fascinating topic.

In summary, autophagy plays a major role in plant adaptation to abiotic stress, regulated under stress at the transcriptional and post-transcriptional levels. Yet, further research regarding the factors inducing stress-related autophagy is required, as well as studies identifying autophagy targets under stress. Moreover, additional investigations regarding the role of autophagy in stress combinations and recovery from stress will help us get closer to an understanding of the role of autophagy in the field. Recent studies suggest that increased autophagic activity can improve plant performance under abiotic stress. These encouraging results, coupled with a greater understanding of autophagy induction, could have far-reaching implications in improving crop resistance to changing environmental conditions.

## Autophagy and immunity

**Figure 5.**
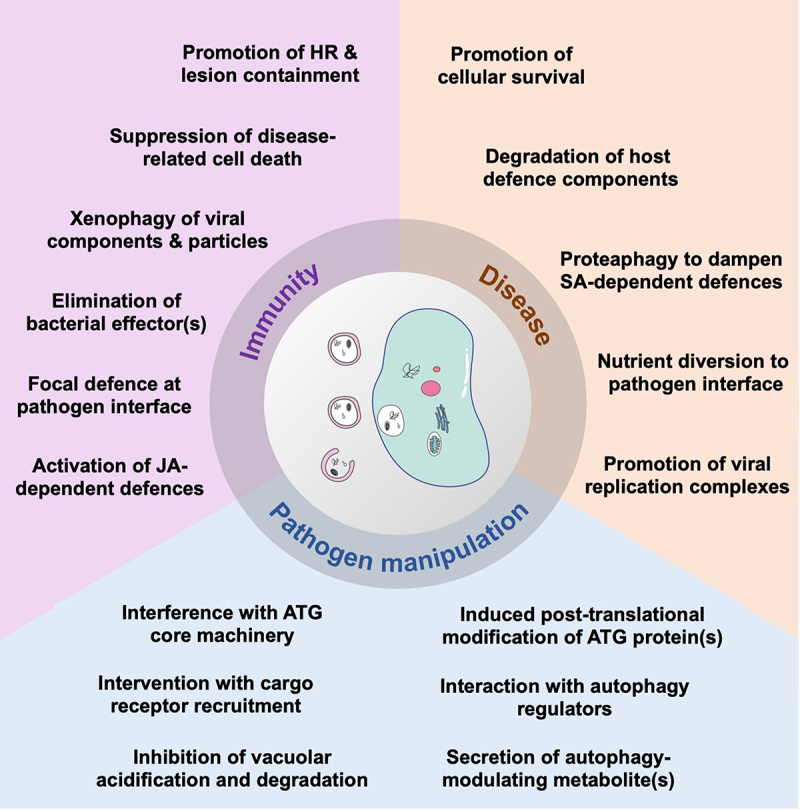
Overview of known functions of autophagy in immunity and disease, and strategies of pathogens to manipulate autophagy processes for their own benefit. Autophagy plays a dual role in the regulation of the immunity-related hypersensitive response (HR) upon infection with various pathogens and suppresses disease-related cell death of necroptrophic fungi. Selective autophagy pathways target viral proteins and particles as well as bacterial effector proteins for degradation. Furthermore, autophagosomes are diverted towards haustoria to mediate focal defence responses against an oomycete pathogen, and autophagy mechanisms are involved in the activation of jasmonic acid (JA)-dependent defences against nematodes. In contrast, cytoprotective functions of autophagy benefit infection by increasing host cell survival, and selective autophagy pathways are hijacked by pathogens to eliminate defence components and to target the proteasome involved in salicylic-dependent immune responses. Autophagic structures are also likely involved in nutrient diversion to the haustorial feeding sites of oomycetes and promote formation of viral replication complexes. Pathogens are able to manipulate autophagy processes by various strategies. These include effector-mediated interactions with and/or modifications of ATG proteins and autophagy regulators as well as interruption of vacuolar functions required for autophagic cargo degradation. Autophagy levels are also modulated by fungal secretion of secondary metabolites. See text for further details.

Research over more than a decade has provided compelling evidence that autophagy plays multifaceted roles in plant immunity and that pathogens have evolved sophisticated measures to manipulate autophagy processes for their own benefit. Pioneering work focused primarily on the functions of autophagy in immunity- and disease-related cell death, revealing both pro-survival and pro-death activities of autophagy in response to pathogens with different lifestyles. Recent efforts showcased the importance of selective autophagy components and pathways as part of the host immune weaponry or, if hijacked by pathogens, as powerful tools for counterdefence and alteration of cell functions. In this section, we will highlight the latest advances in understanding the complex roles of autophagy in plant immunity and disease (Figure 5), and discuss major knowledge gaps and outstanding questions for future research.

### Autophagy and immunity-associated cell death

The plant innate immune system builds on the two interconnected branches of pattern- and effector-triggered immunity (PTI/ETI) to recognize and respond to pathogen challenge (for an detailed overview, see [236]). ETI is activated by intracellular nucleotide-binding, leucine-rich repeat containing (NLR) immune receptors and often associated with a localized PCD reaction, known as the hypersensitive response (HR). Autophagy has previously shown to promote the HR conditioned by certain NLRs during bacterial, oomycete, and viral infection [49,237,238], but is also required to confine the HR and prevent spreading of cell death into healthy tissues [239-241]. Although such dual role of autophagy in HR regulation has long been established in the Arabidopsis and *N. benthamiana* models, the underlying mechanisms and the spatial-temporal control of the opposing activities remain largely unknown. The pro-survival function of autophagy outside of HR lesions has been mainly assigned to its homeostatic role in removing harmful components and attenuating cellular stress associated with salicylic acid (SA)- and NPR1 (non-expressor of pathogenesis-related protein 1)-dependent systemic immune responses [242,243]. In this context, the recently discovered formation of SA-induced NPR1 condensates (SINCs) was shown to directly contribute to cell survival by sequestering ETI- and stress-related components for subsequent degradation [244]. Since the autophagy protein ATG8a and cargo receptor NBR1 co-localise with SINCs, it is tempting to speculate that autophagy processes are involved in either condensate formation and/or turnover. Likewise, it remains to be investigated whether autophagy is engaged in the selective removal of NPR1 and/or other negative PCD regulators at the initial infection site to facilitate HR induction. A pro-death function of autophagy could also be connected to the activation and recycling of immune receptors involved in HR. Based on the recently emerged PTI-ETI crosstalk, this may not only apply to specific NLRs, as discussed before [245], but also to PTI-related pattern recognition receptors like FLS2 (flagellin sensitive 2), which is required for full HR activation upon bacterial infection [246]. Strikingly, FLS2 homeostasis is controlled by autophagy through the selective cargo receptors ORM1 (orosomucoid 1) and ORM2 [247], yet the relevance of these processes for the HR remains to be addressed.

In addition to its catabolic function, two recent reports suggest a novel role of the membrane trafficking activities of autophagy in the dual regulation of cell death. On the one hand, spatial HR restriction in Arabidopsis upon *Pseudomonas syringae* pv. *tomato* (*Pst*) recognition was found to rely on the autophagy-dependent secretory transport of monolignols, which serve as lignin precursors in cell wall lignification resulting in the physical isolation of infection sites [248]. On the other hand, promotion of carbon starvation-induced PCD in potato was shown to involve the autophagy-mediated translocation of the VPE (vacuolar processing enzyme) to the vacuole [249]. VPE exhibits caspase-like activities and is a well-known executioner of developmental and stress-related PCD including the HR in response to tobacco mosaic virus (TMV) and other elicitors [250,251]. However, whether the autophagic trafficking of VPE is indeed decisive for the autophagy dependency of some NLR-triggered HR pathways requires further investigation.

### Autophagy and disease resistance

Consistent with the frequently observed uncoupling of NLR-conditioned HR from growth restriction of (hemi)biotrophic pathogens [252-254], the autophagy-dependent control of immune cell death was found to influence only occasionally ETI-associated disease resistance [49,237]. In contrast, autophagy plays an important role in the defence against necrotrophs, which appears to be tightly linked to the suppression of disease-associated ‘necrotic’ cell death induced by the pathogens to retrieve nutrients from host tissue [41,159,255]. However, the exact mechanisms of defensive autophagy during plant-necrotroph interactions are far from being understood. Previous reports suggested the interplay and/or cooperation of autophagy with hormone and metabolic defence networks [40], for instance through the interaction of ATG18a with WRKY33, a key transcriptional regulator of disease resistance against necrotrophic fungi [40]. Remarkably, the activation of autophagy-dependent ‘immune’ cell death was found to counteract disease progression by *Botrytis cinerea* and *Sclerotinia sclerotiorum* [255,256], supporting the view that autophagy primarily acts during a short (“cryptic”) biotrophic phase required for the pathogenic fungi to initiate growth and establish infection [257]. Likewise, cytoprotective, pro-survival activities of autophagy could negatively impact the transition to necrosis by mediating the selective removal of host- or pathogen-derived compounds associated with cell death activation.

The importance of selective autophagy mechanisms in plant immunity became particularly evident by the analysis of autophagy functions during compatible plant-virus interactions. Similar to the situation in animals, a diverse set of DNA and RNA viruses were shown to be targeted by autophagy as part of the antiviral host response. Thus far, the elimination of certain virulence factors, including various viral suppressors of RNA silencing (VSR) [258-261], a replicase [262] and a movement protein [263], has emerged as the predominant autophagic activity to limit virus infection in plants. In addition, the NBR1-mediated degradation of viral particles and non-assembled capsid protein (P4) of cauliflower mosaic virus (CaMV) highlighted the plant’s capacity to remove an entire intracellular pathogen and/or its structural component [264] (Figure 3F), closely resembling ‘xenophagy’ and ‘virophagy’ pathways in animal cells [265]. Besides NBR1, a few other host proteins (e.g. rgs-CaM [266], P3IP [260], Beclin1/ATG6 [262], and VISP1 [267]) have been implicated as cargo receptors in antiviral autophagy, while some viral proteins were shown to be degraded via direct ATG8-binding [261,263,268].

Notably, NBR1-based selective autophagy has also been linked to the resistance response against virulent bacterial and oomycete infections. NBR1 was found to dampen *Pst* virulence by counteracting the establishment of a pathogen-induced aqueous microenvironment in the apoplast [269]. Yet, it remained unknown whether this anti-bacterial pathway is mediated by the NBR1-dependent degradation of a bacterial effector, such as HopM1, or a specific host protein involved in disease-promoting `water-soaking´. Intriguingly, a recent report demonstrated that NBR1/Joka2 is able to directly target the XopL effector protein from *Xanthomonas campestris* pv. *vesicatoria* (*Xcv*) [270], indicating that NBR1 has evolved as a cargo receptor for the xenophagic degradation of virulence factors across different pathogen classes. In contrast, NBR1-stimulated disease resistance against the oomycete *Phytophthora infestans* has been linked to a focal immune response at the plant-pathogen haustorium interface, which was proposed to rely on the targeted delivery of as yet unknown anti-microbial cargo rather than the removal of secreted effectors [271].

Recently, selective autophay has been also reported to promote jasmonic acid (JA)-dependent resistance against root-knot nematode (RKN) infection [272]. Autophagy stimulation in RKN-challenged tomato plants mediates the selective degradation of the negative JA signalling regulator JAM1 (jasmonate-associated myc2-like 1) via direct interaction with ATG8 proteins. Autophagic removal of JAM1 leads to the activation of the ERF1 transcription factor, which induces JA-dependent defence genes and positively feedbacks on autophagy regulation by enhancing *ATG* gene expression. It remains to be investigated whether autophagy is similarly integrated in the ERF1-branch of the JA signalling pathway during the resistance response against necrotrophic pathogens [273].

### Autophagy manipulation by pathogens

Mounting evidence suggests that pathogens translocate effector proteins or other virulence factors to target the autophagy machinery, its regulators or associated cellular pathways, leading to the activation or suppression of autophagy to the favour of infection. A recent interaction screen with a large collection of effectors from bacterial, fungal, oomycete and nematode pathogens revealed that a significant proportion of the pathogen’s effector repertoire has the potential to interfere with autophagy via direct binding with core ATG proteins [274]. However, the mechanistic details and relevance of these interactions during infection remain to be largely explored. Nonetheless, the initial analysis of three selected *Pst* effectors (HrpZ1, HopF1, AvrPtoB) suggested that both the inhibition and enhancement of autophagy can promote bacterial virulence. In support of this notion, the *Pst* effector HopM1 was previously shown to induce autophagy and proteasome degradation, indicating that *Pst* hijacks the proteaphagy pathway to suppress SA-dependent defence responses for enhanced pathogenicity [269]. On the contrary, the *Xcv* effector XopL inhibits host autophagy by targeting SH3P2, a central regulator of autophagosome biogenesis, to counteract the antibacterial functions of NBR1 [270]. Together, these findings suggest that certain autophagy activities and/or pathways have different functions during the bacterial infection cycle and that effectors are likely secreted in a temporally coordinated fashion to precisely alter autophagy levels for growth promotion.

Due to their intracellular and obligate biotrophic lifestyle, viruses are particularly challenged to activate or maintain beneficial activities of autophagy while evading or suppressing antiviral xenophagy. Several reports have indicated that virus propagation relies on a functional cytoprotective autophagy pathway to dampen disease-related cellular stress and promote host survival [258,259,264]. Besides being activated as a “by-product” of infection and triggered e.g. by SA-mediated defence signalling [259], autophagy can be directly induced and hijacked by viral proteins. For instance, the geminiviral virulence factor βC1 activates autophagy by interfering with the interaction between the negative autophagy regulator GAPC and the core autophagy protein ATG3 [275]. Furthermore, the poleroviral P0 protein triggers an ER-derived autophagy pathway involving the cargo receptors ATI1 and ATI2 to mediate the degradation of AGO1, the central element of the RNA-induced silencing complex (RISC) [133]. Viral proteins were also proposed to induce the selective removal of other host defence components like the cell-to-cell transport regulator REM1 (remorin 1) [276,277] and the silencing pathway component SGS3 [125,278]. In the latter case, cucumber mosaic virus (CMV) co-opts the peptide cargo receptor VISP1 to target the SGS3/RDR6-formed small-interfering RNA bodies for autophagic degradation [125]. Notably, at later stages of CMV infection, VISP1 mediates the turnover of the increasing amounts of the VSR proteins 2b instead of SGS3 [267], which limits viral accumulation and adds to the self-attenuation strategy of the virus to dampen disease severity and enhance plant tolerance [259,279].

In order to cope with autophagy-mediated antiviral defences, viruses have evolved various strategies to subvert autophagic processes. Barley stripe mosaic virus (BSMV), for instance, exploits the γb protein to disrupt the ATG7-ATG8 interaction required for autophagosome formation [280]. In addition, the BSMV γa replicase inhibits vacuolar acidification and autophagosomal degradation through interaction with a subunit of the vacuolar ATPase [281]. A recent report further demonstrated that the C4 protein of the geminivirus cotton leaf curl multan virus (CLCuMuV) interacts with the negative autophagy regulator eIF4A to stabilize its association with ATG5, thus preventing the function of ATG5 in the core autophagy machinery [282]. Turnip mosaic virus (TuMV) utilizes the VPg and 6K2 proteins to block the NBR1-mediated degradation of the silencing suppressor HCpro; however, the exact mechanisms and host targets underlying the inhibitory effect remain to be resolved [258]. Strikingly, the interference of TuMV with NBR1 flux seems to be accompanied by the viral replicase (NIb)-mediated recruitment of NBR1 and its interacting ATG8f isoform for the formation of viral replication complexes on the tonoplast [283].

The effector-induced inhibition of NBR1-dependent autophagy defences and diversion of autophagic processes for microbial pathogenesis are also illustrated during infection with *Phytophthora infestans*. The pathogen deploys the RXLR effector PexRD54 to specifically bind the potato isoform ATG8CL, thereby outcompeting Joka2/NBR1 and, presumably, its antimicrobial cargo from ATG8CL-containing complexes [271,284]. Intriguingly, PexRD54 stimulates autophagosome formation at haustoria by recruiting the vesicle trafficking regulator Rab8a to ATG8CL structures. PexRD54-mediated autophagy activation resembles a carbon starvation-induced autophagy response and is assumed to redirect plant nutrients and/or other resources to the pathogen interface for growth and proliferation [285].

Due to the importance of autophagy in the resistance response against necrotrophic fungi, the capacity of these pathogens to subvert autophagy during early stages of infection likely influences disease progression. However, surprisingly little is known about fungal strategies to interfere with autophagy processes. For instance, the secretion of oxalic acid by *S. sclerotiorum* was shown to induce necrotic cell death by the suppression of autophagy and autophagy-dependent hypersensitive cell death [256], yet the molecular details of this interplay remain to be investigated. Similarly, autophagy is suppressed during *B. cinerea* infection upon phosphorylation of the autophagy protein ATG18a by the receptor kinase BAK1 [41], a key regulator of PRR-mediated immune signaling. Whether this effect is triggered by fungal virulence factors is unclear, but it is tempting to speculate that several necrosis-inducing effectors (e.g. *Botrytis* BcXYG1 [xyloglucanase 1]) hijack the recognition by BAK1 and/or associated receptor complexes to overcome autophagy-mediated defences for cell death and disease promotion [286,287].

Finally, a recent study showed that an effector from parasitic cyst nematodes (Heterodera and Globodera ssp.), termed NMAS1 (nematode manipulator of autophagy system 1), interacts with ATG8 proteins to suppress immunity-related ROS production [288]. Together with the enhanced resistance of *atg* mutants to *Heterodera schachtii* infection [288], these findings suggest that cyst nematodes require autophagy to promote disease, which is strikingly different from the positive role of autophagy in the resistance against root-knot nematodes [272].

In summary, numerous studies have now demonstrated the enormous complexity of the autophagy-pathogen interplay which is in particular reflected in the often overlapping operation of autophagy defences, their effector-mediated subversion and co-option of autophagic components for microbial pathogenesis (Figure 5). Hence, future research faces the main challenge to untangle the specific mechanisms underlying the pro- and antimicrobial autophagic activities and to distinguish them from more general functions of autophagy in cellular homeostasis and stress tolerance. To achieve this, advanced tools and methods need to be further developed to allow e.g. (i) the assessment of autophagy processes at spatio-temporal resolution, (ii) the systematic search for autophagy-related effector targets, (iii) the comprehensive inventory of the selective receptor repertoire and autophagic cargoes, and (iv) the analysis of autophagy processes beyond their involvement in canonical catabolic functions. While most of these approaches will likely continue to explore well-established pathosystems in the Arabidopsis and *N. benthamiana* models, an increasing attention has recently been directed to the analysis of autophagy interactions with economically relevant pathogens in crop species [289,290]. Such research will add to the growing interest in translational approaches to assess the potential of autophagy modulation for agricultural trait development including disease resistance.

## Autophagy in crops

### Genome-wide identification of ATG genes in crop species

In contrast to yeast, which typically have only one gene encoding each ATG protein, plants generally have a few to several isoforms of core ATG components (Table 1). While the precise number of ATG isoforms may vary among plant genomes depending on the quality of their gene annotations, the largest ATG families in crop species are ATG8 and ATG18. The ATG8 protein family is represented by four isoforms in foxtail millet *(Setaria italica*), five in maize (*Zea mays*), seven in rice (*Oryza sativa*), 13 in wheat (*Triticum aestivum*), eight in alfalfa (*Medicago truncatula*), 14 in cabbage (*Brassica oleraceae)*, 22 in rapeseed (*Brassica napus)*, and nine in *Arabidopsis thaliana* (AtATG8a-i) [291,292], whereas the ATG18 family consists of four isoforms in wheat, six in rice, eight in alfalfa, cabbage, and Arabidopsis (AtATG18a-h), and ten in maize [293-295], with one study reporting 31 ATG18 isoforms in maize [296].

**Table 1.** Number of *ATG* genes identified in different plant species, including several major crops.

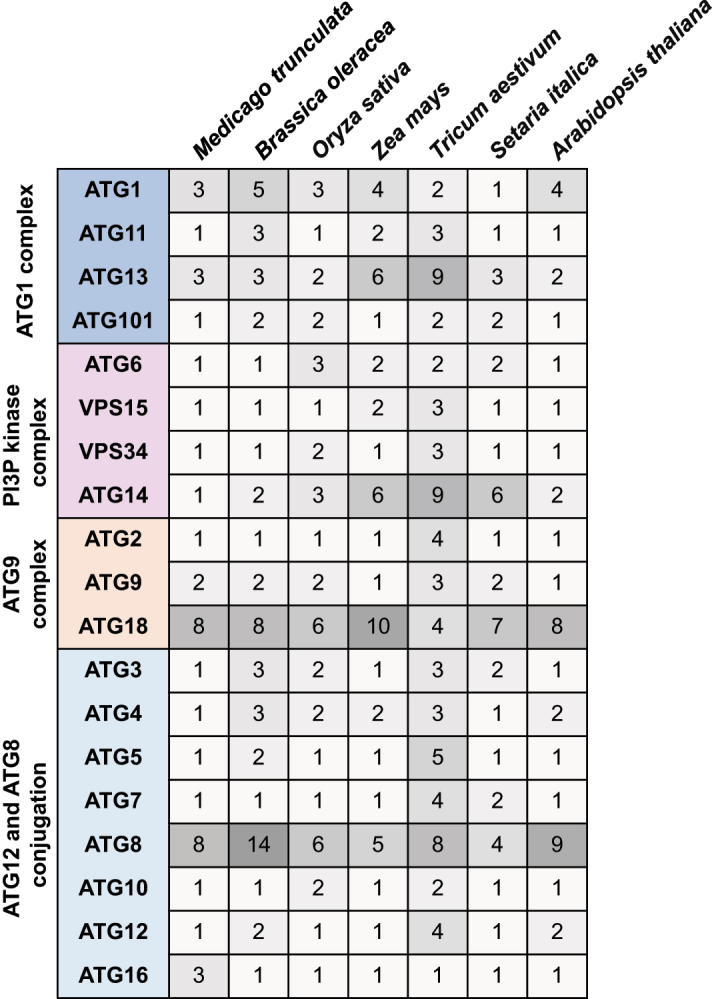

The expansion and diversification of ATG8 isoforms have occurred repeatedly in plant lineages. [291]. A phylogenetic analysis on 59 plant genomes showed that plant ATG8 isoforms group into two major clades: clade I (including AtATG8a-g) with similarities to fungal ATG8 proteins, and clade II (comprising AtATG8h and i) with similarity metazoan ATG8 homologs [102,291]. Both clades have well-supported subclades specific to plant groups, including Brassicales, Solanaceae, and Poaceae (including all cereals) [291]. The ATG8 family is believed to have expanded through functional specialization, although the specific roles of individual plant ATG8 proteins remain largely unknown. In wheat, TaATG8a, g (clade I), and h (clade II) are upregulated in response to a wide range of abiotic stressors, including high salt, drought, low temperature/darkness, and nutrient deficiency. However, TaATG8a is particularly responsive to high salinity, as shown by its most pronounced upregulation under this stress condition [297]. Consistently, overexpression of TaATG8a alone confers enhanced tolerance to high salt in wheat [292], suggesting a specific role for this isoform in salt stress response. Further supporting the notion that plant ATG8 isoforms have evolved specific function, the silencing of one of the 13 ATG8 isoforms in wheat, TaATG8j (clade I, clustering close AtATG8a), is enough to enhance susceptibility to stripe rust [298].

The ATG18 family in Arabidopsis consists of eight isoforms (ATG18a-h), which are grouped into three subfamilies based on the presence of different structural motifs, and are believed to be present in all vascular plants [296]. In Arabidopsis, ATG18a plays a role in autophagosome formation in response to starvation and senescence [299] as well as in the turnover of the ER through a process called ERphagy [42]. *ATG18a* is expressed in response to nitrogen (N) starvation whereas both *ATG18a* and *f* are upregulated in seedlings transferred to sucrose-lacking medium [299]. In tomato (*Solanum lycopersicum*), *ATG18a, b*, and *f* are upregulated in response to drought and *ATG18f* is required for autophagosome formation under drought conditions [19]. In common bean (*Phaseolus vulgaris*), the expression of the eight ATG18 isoforms varies among tissues and between nodulated and mycorrhized roots, suggesting functional specialization [296].

As mentioned above, the overexpression of ATG18a in apple *(Malus domestica)* seems enough to confer enhanced protection against both biotic and abiotic stresses [209,300]. The fact that a single isoform can confer enhanced resistance to various stresses is remarkable, considering the coordination required among numerous ATG components to execute autophagy. These findings highlight the crucial roles of ATG8 and ATG18 subunits and make them prime candidates for breeding and genetic engineering programs aimed at generating stress-tolerant plants.

The ATG8 and ATG18 protein families are well-known for their large and diversified nature within vascular plants. However, some cereal crops have also shown an expansion in other ATG components. For instance, while Arabidopsis, rice, and alfalfa have two or three ATG13 isoforms, maize and wheat contain six and nine ATG13 proteins, respectively [301]. The complexity of the wheat genome can explain the high number of *ATG* genes due to two hybridization events and massive local rearrangement. However, it is unknown whether the multiple ATG13 isoforms have specific functions or are entirely redundant.

Although the molecular and cellular roles of many ATG components have been initially characterized in *Arabidopsis thaliana*, studies in crop species have revealed the crucial function of autophagy in grain yield [302,303], biotic stress tolerance [304], nutrient recycling [302,305-307], fruit ripening [155], programmed cell death in cereal grain pericarps [308], and overall crop fitness. Below we discuss some relevant studies related to the role of autophagy in N recycling, crop yield, and development.

### Autophagy during nitrogen remobilization in crops

Nutrient recycling through cellular catabolic pathways is important for crop yield [309], particularly under nutrient-limiting conditions. As autophagy is a key cellular recycling pathway, understanding how it is modulated is fundamental to enable sustainable production of cost-competitive crops by better understanding nutrient recycling and increasing yields in low-nutrient soils while minimizing fertilizer requirements. Several studies have focused on how autophagy controls N recycling. Approximately 75% of the N present in a plant is in the leaf chloroplasts [310], with Rubisco constituting between 20 and 50% of the total leaf protein content [311,312]; thus turnover of chloroplasts and other organelles to mobilize assimilated N from old vegetative tissues into new growing organs and seeds during leaf senescence is important for crop yield [313]. Given that, as indicated by ^15^N flux experiments [180], autophagy plays the major role in remobilizing nitrogen captured in Rubisco it is perhaps unsurprising that autophagy deficient mutants are hypersensitive to N deficiency [180,182,314-316].

In maize, autophagy is activated under N-limiting conditions and during senescence, as evidenced by the accumulation of lipidated forms of ATG8 in leaf tissues [317]. Maize *atg12* mutants with reduced ATG8 lipidation develop normally but produce smaller seeds even when grown under nutrient-rich conditions. This is consistent with reduced N mobilization from vegetative tissues into seeds in the *atg12* mutant, as demonstrated by ^15^NO_3_^−^ labeling experiments [307]. Under limited N availability, growth of maize *atg12* mutant plants is severely reduced with enhanced leaf senescence [307].

Multi-omic analyses performed in maize *atg12* mutant plants under either N-rich or N-deficient conditions revealed some intriguing and unanticipated insights. For example, although *atg12* plants grow normally when N is available, their proteomic, transcriptomic, and metabolic profiles are drastically altered compared to wild type, suggesting a deep reprograming needed to overcome the lack of macroautophagy. The leaves of *atg12* mutant plants either under N-rich or N-low conditions, showed drastic changes in metabolic profiles related to fatty acid catabolism, including free fatty acids, lysolipids, oxylipins, glycerolipids, phospholipids, sphingolipids, and galactolipids, suggesting that other catabolic pathways increased lipolysis and membrane turnover when autophagy is impaired [305].

In rice, plants lacking OsATG7 function showed reduced biomass production even when grown under N-rich conditions and reduced N remobilization compared to wild type plants [318]. Among the eight OsATG8 isoforms in rice, *OsATG8b* is strongly upregulated in vegetative tissues during seed development and under N deficiency, consistent with a role in N remobilization. Rice plants with reduced or null expression of *OsATG8b* display reduced vegetative growth and seed yield, while overexpression of *OsATG8b* results in increased seed production [302,319]. Moreover, a ^15^NO_3_^−^ partition analysis confirms the role of OsATG8b in N mobilization from vegetative tissues into seeds [302,319]. Although disruption of OsATG8b function is enough to reduce plant fitness, suggesting reduced or no redundancy with other OsATG8 isoforms, the overexpression of *OsATG8c* in rice is also able to enhance N mobilization with higher grain yields and plant biomass [320], with consistent phenotypes observed by the overexpression of *SiATG8a* in foxtail millet [321] and *CsATG8a* in tea [322].

In barley, the expression of several *ATG* genes (*e.g. HvATG3, HvATG5, HvATG7, HvATG8a, HvATG8c, HvATG9, HvATG18f*) increases during senescence and nutrient mobilization from the flag leaf, which is critical for grain filling [323,324]. This further supports the role of autophagy in N recycling and grain filling in cereals.

### Autophagy in crop development

Autophagy plays diverse roles in the development of different plant species, as revealed by various mutant studies. Mutations in *ZmATG12* lead to reduced seed size without impairing vegetative growth or reproductive development under nutrient-rich conditions [307]. Similarly, mutations in *AtATG7* and *AtATG5*, which also participate in ATG8 lipidation in Arabidopsis, do not block reproductive development [325]. However, in rice, mutations in *OsATG7* cause abnormal pollen development, male sterility, reduced pollen germination, and impaired anther dehiscence due to reduced autophagic activity in the tapetum, abnormal lipid metabolism, and reduced phytohormone content (gibberellins and cytokinins) in anthers [326,327]. In tobacco (*Nicotiana tabacum*), silencing of *NtATG2*, *NtATG5*, and *NtATG7* drastically reduce pollen germination. Pollen grains with silenced *ATG* genes show normal lipid and mitochondrial metabolism and fail to degrade a cytoplasmic layer located under the pollen wall germination aperture, which might need to be removed via autophagy for successful extrusion of the vegetative cell and formation of the pollen tube [328].

As mentioned above, maize *atg12* mutant plants develop smaller seeds even when plants are grown under nutrient rich conditions, suggesting that autophagy may play additional roles in seed development besides nutrient recycling. For instance, maize plants lacking Rtn2 proteins, which are highly expressed in the endosperm and act as selective autophagy receptor for ERphagy, show exacerbated ER stress in aleurone cells of the endosperm [127], suggesting that autophagy may be required during endosperm development to mitigate ER stress during the intense synthesis of storage proteins. However, based on the analysis of *atg12* mutants, autophagy does not seem to be required for programmed cell death in the maize endosperm [307]. The delivery of storage proteins into vacuoles of aleurone cells is mediated by microautophagy [329] but this autophagic process is not dependent on the ATG8 conjugation pathway [307]. Rice plants lacking *Osatg7* produce seeds with extremely low germination rate, chalky appearance, smaller starch granules, and abnormal sugar metabolism [302], while in wheat, silencing of ATG8 results in delayed PCD, increased pericarp thickness, premature seed maturation, and smaller grains [308].

The differences in developmental phenotypes (pollen development and viability; seed and fruit development) seen in different plant species deficient in autophagy are likely due to different functional specializations and requirements for autophagy. Thus, the genetic and molecular analysis of more specific ATG components across plant species will be critical to understand the multiple roles of autophagy in crop yield and fitness.

## Perspectives

The field of plant autophagy is expanding rapidly. However, while the conserved autophagy machinery is well-documented, its interaction with the endomembrane system, selective catabolic pathways, hormonal signaling, and developmental roles together with autophagy’s crucial function in carbon and nitrogen starvation, requires further exploration which should include inducible manipulations of autophagy. Moreover, interactions between autophagy and hormonal signaling pathways, potentially using advanced techniques like tissue-specific knockout mutants, will be essential to clarify specific roles of autophagy in diverse developmental programs.

In the context of abiotic stress, Arabidopsis *atg* mutants’ heightened sensitivity versus enhanced resistance upon ATG overexpression underscore the significance of autophagy in agricultural solutions amid climate change. Recent findings also indicate that pathogens, including bacteria, fungi, oomycetes, and nematodes, manipulate autophagy through direct binding with core ATG proteins. However, mechanistic details and the relevance of these interactions during infection are yet to be fully explored. Nevertheless, unraveling autophagy induction mechanisms are crucial frontiers and has the potential to contribute significantly to advancements in agriculture with new ideas for agronomic interventions.

## List of abbreviations

ARR: *Arabidopsis thaliana* response regulator
ABA: abscicic acid
ABI: ABA insensitive
ACBP3: acyl-Co-A binding protein 3
ACC: 1-aminocyclopropane-1-carboxylate
AGO1: argonaute 1
AIM: ATG8-interacting motif
AMSH3: associated molecule with the SH3 domain of STAM 3
AOX: alternative oxidase
ARF: Auxin responsive factor
ATG: Autophagy-related
ATI: ATG8 interacting protein
BAK1: BRI1-associated kinase 1
BSMV: barley stripe mosaic virus
BES1: BRI1-EMS suppressor 1
BIN2: brassinosteroid insensitive 2
BR: brassinosteroid
BRI1: brassinosteroid insensitive 1
BZR1: brassinazole resistant 1
CAS31: cold-acclimation-specific 31
CaMV: cauliflower mosaic virus
CDC48: cell devision cycle 48
CFS1: cell death related endosomal FYVE/SYLF protein 1
CHMP1: charged multivesicular body protein 1
CML24/38: calmodulin-like 24/38
CMV: cucumber mosaic virus
COST1: constitutively stressed 1
CLCuMuV: cotton leaf curl multan virus
COPII: coat protein complex II
EACS: ER-autophagosome membrane contact site
EE: early endosome
EPCS: ER-PM contact site
ER: endoplasmic reticulum
ERGIC: ER–Golgi intermediate compartment
ERF: ethylene responsive element binding factor
ESCRT: endosomal sorting complex required for transport
E-syts: extended synaptotagmins
ETI: effector-triggered immunity
FER: feronia
FLS2: flagellin sensitive 2
FLZ: FCS-like zinc finger
FMT: friendly
FREE1: FYVE domain protein required for endosomal sorting 1
GA: golgi apparatus
GABA: γ-aminobutyric acid
GABARAP: GABA type A receptor-associated protein
GAPC: cytosolic glyceraldehyde-3-phosphate dehydrogenase
GSNOR: S-nitroglutathione reductase
GSK3: glycogen synthase kinase 3
HDA9: histone deacetylase 9
HR: hypersensitive response
H_2_S: hydrogen sulfide
HY5: elongated hypocotyl 5
IM: ER isolation membrane
ILV: intralumenal vesicle
IRE1: inositol-requiring enzyme 1
JA: jasmonic acid
JAM1: jasmonate-associated myc2-like 1
LE: late endosome
LR: lateral root
LUX: lux arrhythmo
MCS: membrane contact site
MPK3: mitogen-activated protein (MAP) kinase 3
MSBP1: membrane steroid binding protein 1
MVB: multivesicular body
NBR1: neighbor of brca1
NLR: nucleotide-binding, leucine-rich repeat containing
NMAS1: nematode manipulator of autophagy system 1
NPR1: non-expressor of pathogenesis-related protein 1
NUE: nutrient use efficiency
ORM: orosomucoid
ORP2A: oxysterol-binding protein related protein 2A
PAS: preautophagosomal structure/phagophore assembly site
PCD: programmed cell death
PE: phosphatidylethanolamine
PI3P: phosphatidylinositol 3-phophate
PI3K: phosphatidylinositol 3-kinase
PM: plasma membrane
*Pst*: *Pseudomonas syringae* pv. tomato
PTI: pattern-triggered immunity
PUX: plant UBX domain-containing protein
PVC: prevacuolar compartment
RDR6: RNA-dependent RNA polymerase 6
REM1: remorin 1
RISC: RNA-induced silencing complex
RKN: root-knot nematode
ROS: reactive oxygen species
RPN10: regulatory particle non-ATPase 10
Rtn1: reticulon 1
SA: salicylic acid
SAR1: secretion associated RAS 1
SGS3: suppressor of gene silencing 3
SH3P2: SH3 domain-containing protein 2
SINAT: seven in absentia of *Arabidopsis thaliana*
SINCs: SA-induced NPR1 condensates
SNARE: N-ethylmaleimide-sensitive factor attachment protein receptor
SnRK1: snf1-related kinase 1
SOC1: suppressor of overexpression of constans 1
STX17: syntaxin 17
TGA9: TGACG motif-binding protein 9
TGN: trans-Golgi network
TOC: translocon at the outer envelope membrane of chloroplasts
TRAF: tumor necrosis factor receptor-associated factor
TOC1: timing of cab expression 1
TOPP: type one protein phosphatase
TORC: target of rapamycin complex
TPC: TPLATE complex
TuMV: Turnip mosaic virus
TYLCV: tomato yellow leaf curl virus
UBAC2A/B: ubiquitin-associated protein 2A/B
UDS: ubiquitin docking site
UFM1: ubiquitin fold modifier 1
UIM: Ubiquitin interacting motif
VAMP: vesicle-associated membrane protein
VAP27: VAMP-associated protein 27
VISP1: virus induced small peptide 1
VMP1: vacuolar membrane protein 1
VPE: vacuolar processing enzyme
VPS23A: vacuolar protein sorting 23A
VPS34: vacuolar protein sorting 34
VSR: viral suppressors of RNA silencing
VTI12: vesicle transport v-SNARE 12
*Xcv*: *Xanthomonas campestris* pv. vesicatopria
XYG: xyloglucanase 1
